# B Cells from Aged Mice Do Not Have Intrinsic Defects in Affinity Maturation in Response to Immunization

**DOI:** 10.4049/jimmunol.2300318

**Published:** 2023-09-27

**Authors:** Jia Le Lee, Silvia Innocentin, Alyssa Silva-Cayetano, Stephane M. Guillaume, Michelle A. Linterman

**Affiliations:** Immunology Program, Babraham Institute, Babraham Research Campus, Cambridge, United Kingdom

## Abstract

Affinity maturation, the progressive increase in serum Ab affinity after vaccination, is an essential process that contributes to an effective humoral response against vaccines and infections. Germinal centers are key for affinity maturation, because they are where B cells undergo somatic hypermutation of their Ig genes in the dark zone before going through positive selection in the light zone via interactions with T follicular helper cells and follicular dendritic cells. In aged mice, affinity maturation has been shown to be impaired after immunization, but whether B cell–intrinsic factors contribute to this defect remains unclear. In this study, we show that B cells from aged BCR transgenic mice are able to become germinal center B cells, which are capable of receiving positive selection signals to a similar extent as B cells from young adult mice. Consistent with this, aging also does not impact the ability of B cells to undergo somatic hypermutation and acquire affinity-enhancing mutations. By contrast, transfer of B cells from young adult BCR mice into aged recipients resulted in the impaired acquisition of affinity-enhancing mutations, demonstrating that the aged microenvironment causes altered affinity maturation.

## Key Points

- Aged GC B cells can undergo positive selection in young mice upon immunization.
- Aged GC B cells acquire high-affinity mutations postimmunization in young mice.
- B cells from young mice have fewer high-affinity mutations in aged hosts.

## Introduction

Affinity maturation, the progressive increase in the affinity of serum Abs over time, is an important process that underlies an effective humoral response against vaccines and infections (1, 2). Germinal centers (GCs) are the cellular engines of affinity maturation. Within GCs, B cells undergo somatic hypermutation of their Ig genes in the dark zone, which generates a pool of B cells carrying random mutations that will then undergo selection in the light zone (3). B cells carrying functional BCRs that can uptake and present Ag to T follicular helper (Tfh) cells will receive positive selection signals, which induce upregulation of the proto-oncogene cMyc and promote cyclic re-entry in the dark zone for further somatic mutations and clonal expansion (4–6). Eventually, these B cells exit the GC as memory B cells or long-lived Ab-secreting plasma cells, which are key in conferring protection against future infections.

The impaired vaccine response during aging has been widely characterized across different vaccine formulations. This defect involves not only a quantitative reduction in vaccine-specific Ab titers (7–9) but also a higher incidence of nonspecific autoantibodies (10) and reduced adaptability of B cell response to drifted virus strains in older individuals (11). Analysis of the Ig genes of GC B cells from the Peyer’s patches and spleens of older people revealed that the mechanism of somatic hypermutation is unaltered with age (12). However, in vivo studies in mice show that affinity maturation is impaired with age in response to immunization, as shown by fewer high-affinity GC B cells and fewer Ag-specific plasma cells in the bone marrow of aged mice postvaccination (13–15). This age-related defect in GC response and output is a result of a reduction in GC magnitude, as well as impairment in the selection process (13–16).

Because an effective humoral response during vaccination relies on the coordinated interaction of multiple cell types, a multitude of factors can contribute to age-related defects in vaccine responses. Previous studies have shown that B cells from older people and aged mice have no intrinsic defects in responding to stimulation and differentiating into plasma cells (13, 17, 18). However, whether there are B cell–intrinsic defects in the process of affinity maturation remains unclear. In this study, we tracked the response over time of Ag-specific B cells derived from 6- to 12-wk-old young adult and >90-wk-old aged B1-8i transgenic (Tg) mice transferred into young wild-type (WT) recipient mice after immunization. B cells from aged mice had no defects in undergoing class-switch recombination, or in becoming GC B cells or plasmablasts, compared with those from young adult mice. We also show that B cells derived from aged B1-8i mice were equally able to upregulate cMyc in the GC, suggesting no intrinsic defects in their ability to receive positive selection signals. Sequencing of the V_H_186.2 H chain region of 4-hydroxy-3-nitrophenylacetyl (NP)-specific GC B cells derived from young and aged B1-8i mice revealed no age-related intrinsic defects in the rate of somatic hypermutation or their ability to acquire the affinity-enhancing W33L mutation. Conversely, NP-specific GC B cells from young donor B1-8i mice displayed defects in somatic mutation frequency and the acquisition of the W33L mutation when transferred into aged recipient mice. This highlights that the poor quality of the GC response in aging is not due to B cell–intrinsic impairments. Rather, B cell–extrinsic factors are bigger contributors to age-related impairments in affinity maturation in response to immunization.

## Materials and Methods

### Mouse husbandry and maintenance

**FIGURE 1.**
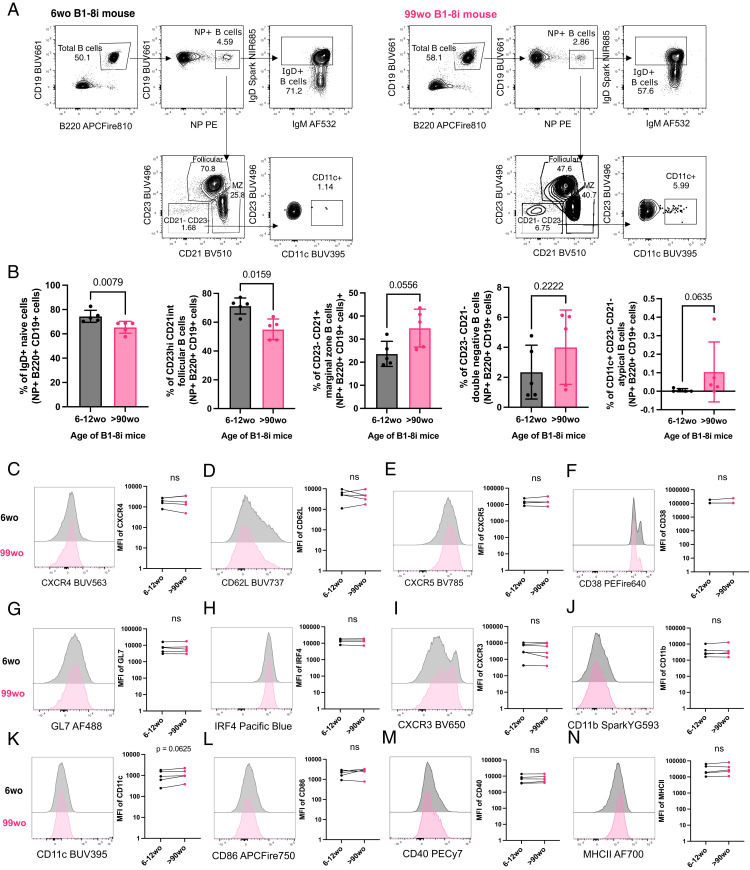
NP-specific B cells from aged mice are phenotypically similar to those from young adult mice. (**A**) Gating strategy for NP-specific IgD^+^ B cells, follicular B cells (CD23^hi^CD21^int^), marginal zone B cells (CD23^−^CD21^+^), CD23^−^CD21^−^ cells, and atypical B cells (CD23^−^CD21^−^CD11c^+^) in young (6 wo) and aged (99 wo) B1-8i Tg mice. Numbers adjacent to gates indicate percentage of parent population. Cells were pregated for live cells and single cells. (**B**) Graphs depicting the percentage of IgD^+^ cells, follicular cells (CD23^hi^CD21^int^), marginal zone cells (CD23^−^CD21^+^), CD23^−^CD21^−^ cells, and CD11c^+^CD23^−^CD21^−^ cells among NP^+^B220^+^CD19^+^ cells in young (6–12 wo) and aged (90–100 wo) B1-8i Tg mice. Bar height corresponds to the mean, error bars indicate SD, and each symbol represents values from individual mice. Statistics were calculated using unpaired Mann–Whitney *U* test. Data were pooled from five independent repeat experiments. (**C**–**N**) Representative flow cytometric histograms and graphs showing the mean fluorescence intensities (MFIs) of CXCR4 (C), CD62L (D), CXCR5 (E), CD38 (F), GL7 (G), IRF4 (H), CXCR3(I), CD11b (J), CD11c (K), CD86 (L), CD40 (M), and MHC class II (N) of NP^+^B220^+^CD19^+^ cells from young (6 wo) or aged (99 wo) B1-8i Tg mice. Each symbol represents values from individual mice, with young and aged donors from the same experiment shown as paired values. Statistics were calculated using Wilcoxon matched pairs signed rank test. Data are pooled from at least four independent repeat experiments. wo, wk old.

**FIGURE 2.**
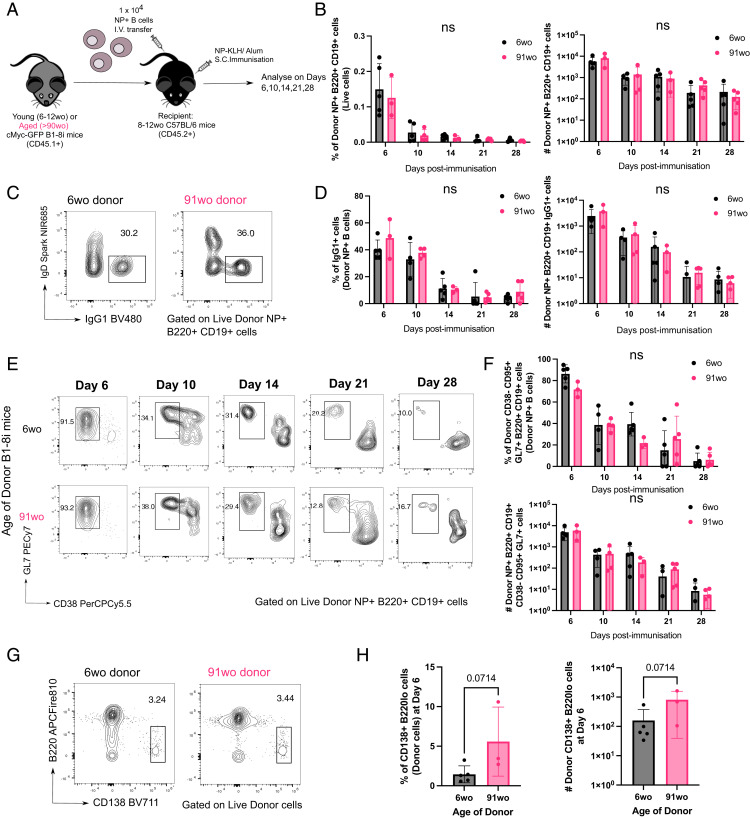
B cells from aged donor mice have no defects in class-switch recombination, entering the GC response and plasmablast differentiation. (**A**) Schematic diagram of adoptive transfer experiments to compare the response of B cells from young (6–12 wo) and aged (>90 wo) mice in young recipient mice postimmunization with NP-KLH in Alum. Draining iLNs were taken at indicated times postimmunization for downstream analyses. (**B**) Graphs depicting the percentage and number of donor NP^+^B220^+^CD19^+^ cells out of live cells in recipient iLNs at different time points posttransfer and immunization. (**C**–**F**) Representative flow-cytometric plots showing gating strategies for donor-derived IgG1^+^IgD^−^ B cells (C) and CD38^−^GL7^+^ GC B cells (E) from 6-wo or 91-wo donor mice at different time points posttransfer and immunization. Numbers adjacent to gates indicate percentage of donor NP^+^B220^+^CD19^+^ cells. Graphs depicting the percentage and number of donor NP^+^IgG1^+^ cells (D) and donor NP^+^CD38^−^CD95^+^GL7^+^ cells (F) in recipient iLNs at different time points posttransfer and immunization. Statistics were calculated using two-way ANOVA with Sidak’s multiple comparisons test. (**G**) Representative flow-cytometric plots showing gating strategies for donor-derived CD138^+^B220^lo^ cells from 6-wo or 91-wo donor mice at day 6 posttransfer and immunization. Numbers adjacent to gates indicate percentage of donor cells. (**H**) Graphs depicting the percentage and number of donor CD138^+^B220^lo^ cells in recipient iLNs at day 6 posttransfer and immunization. Bar height corresponds to the mean, error bars indicate SD, and each symbol represents values from individual recipient mice. Statistics were calculated using unpaired Mann–Whitney *U* test. Data are representative of two independent repeat experiments. wo, wk old.

**FIGURE 3.**
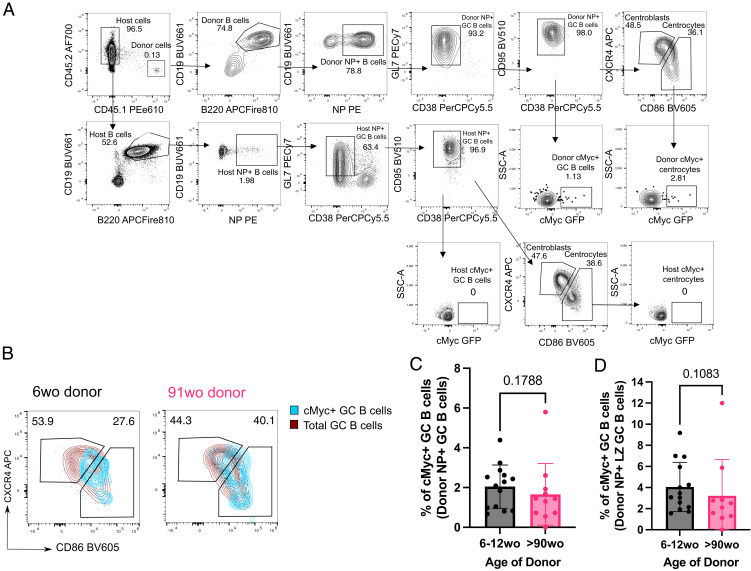
GC B cells from aged donor mice have no intrinsic defects in cMyc upregulation. (**A**) Representative flow cytometric plots showing the gating strategy for cMyc^+^ cells from donor GC B cells (CD45.1^+^CD19^+^B220^+^NP^+^CD38^−^GL7^+^CD95^+^) and LZ (CXCR4^lo^CD86^hi^) GC B cells. (**B**) Flow cytometric plots showing the overlay of cMyc^+^ GC B cells (blue) on total GC B cells (red) gated for dark zone (CXCR4^+^CD86^lo^) and light zone (CXCR4^lo^CD86^+^) phenotype from young adult or aged B1-8i donor mice. Numbers adjacent to gates indicate percentage of donor NP^+^B220^+^CD19^+^CD38^−^GL7^+^CD95^+^ GC B cells. (**C** and **D**) Graphs depicting the percentage of cMyc^+^ GC B cells out of donor NP^+^ GC B cells (C) and donor NP^+^ LZ GC B cells (D) in recipient iLNs at day 6 posttransfer and immunization. Bar height corresponds to the mean, error bars indicate SD, and each symbol represents values from individual recipient mice. Statistics were calculated using unpaired Mann–Whitney *U* test. Data were pooled from three independent repeat experiments.

**FIGURE 4.**
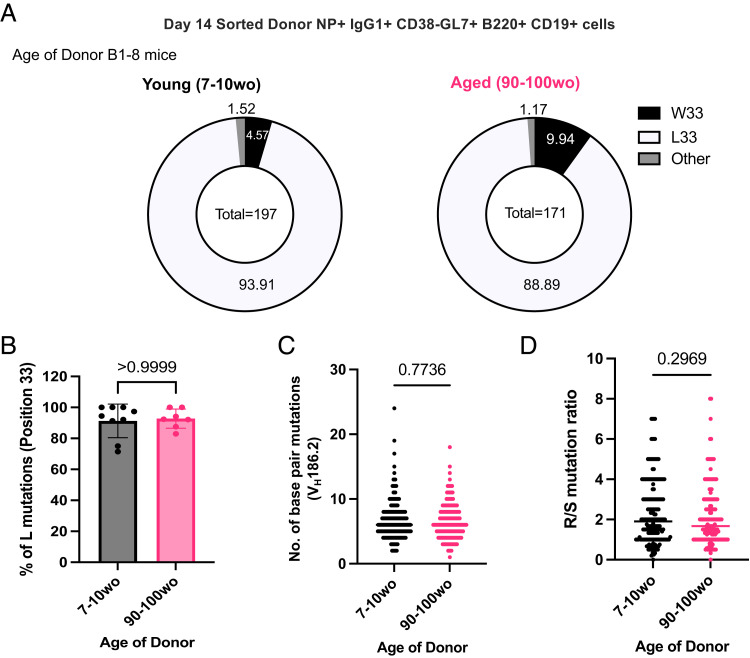
NP-specific B cells from aged mice undergo mutation and selection of high-affinity clones. (**A**) Pie charts indicating the frequency of the affinity-inducing mutation W33L in the CDR1 of V_H_186.2 sequenced from single-cell-sorted NP^+^IgG1^+^CD38^−^GL7^+^B220^+^CD19^+^ cells from either young adult (7–10 wo) or aged (90–100 wo) B1-8i mice in iLNs of young adult (8–12 wo) recipient mice 14 d postimmunization with NP-KLH in Alum. The values in the center of the pie charts indicate the total number of cells sequenced per group (*n* = 7–9 mice per group from two independent experiments, with an average of 20 GC B cells sequenced per mouse). The number of sequences analyzed per recipient mouse is shown in Supplemental Fig. 3A. (**B**) Graph depicting the percentage of sorted GC B cells with the W33L mutation from young adult (7–10 wo) or aged (90–100 wo) B1-8i mice. Bar height corresponds to the mean, error bars indicate SD, and each symbol represents values from individual recipient mice. (**C** and **D**) Graphs depicting the number of single-base-pair mutations (C) and the ratio of replacement: silent mutations (D) in the CDR1 of V_H_186.2 among sorted GC B cells derived from young adult (7–10 wo) or aged (90–100 wo) mice at day 14 postimmunization. Each symbol represents values from a single sorted GC B cell from seven to nine recipient mice per group. Statistics were calculated using unpaired Mann–Whitney *U* test. Data are pooled from two independent repeat experiments. wo, wk old.

**FIGURE 5.**
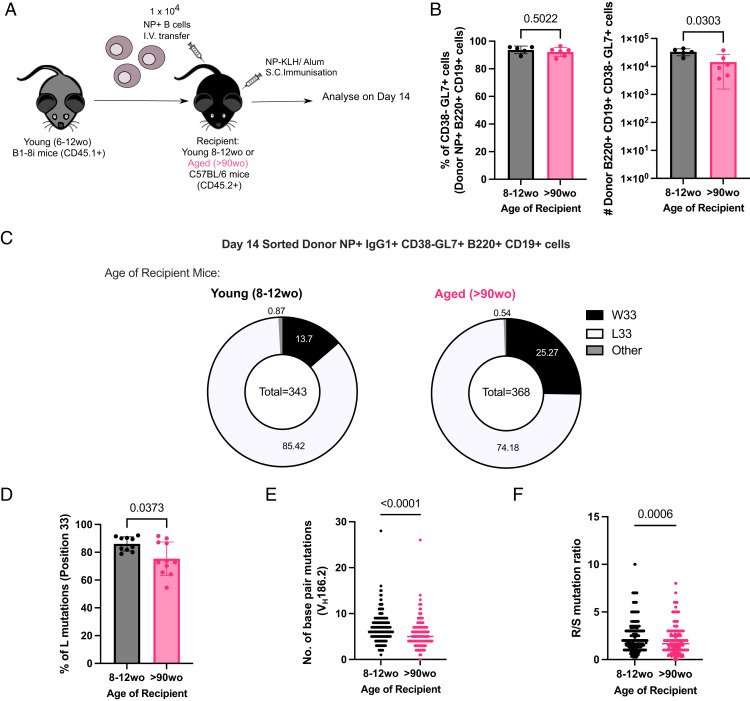
NP-specific B cells from young mice have defects in affinity maturation when transferred into aged recipient mice. (**A**) Schematic diagram of adoptive transfer experiments to compare response of B cells from young (6–12 wo) B1-8i mice in young (8–12 wo) or aged (>90 wo) recipient mice postimmunization with NP-KLH in alum. Draining iLNs were taken at day 14 postimmunization for downstream analyses. (**B**) Graphs depicting the percentage and number of donor NP^+^CD38^−^CD95^+^GL7^+^ GC B cells in recipient iLNs at day 14 posttransfer and immunization. (**C**) Pie charts indicating the frequency of the affinity-inducing mutation W33L in the CDR1 of V_H_186.2 sequenced from single-cell-sorted NP^+^IgG1^+^CD38^−^GL7^+^B220^+^CD19^+^ cells from young adult B1-8i mice in recipient iLNs 14 d postimmunization. The values in the center of the pie charts indicate the total number of cells sequenced per group (*n* = 10–11 mice per group from two independent experiments, with an average of 35 GC B cells sequenced per mouse). The number of sequences analyzed per recipient mouse is shown in Supplemental Fig. 3B. (**D**) Graph depicting the percentage of sorted GC B cells from young adult B1-8i mice with the W33L mutation in young or aged recipients. Bar height corresponds to the mean, error bars indicate SD, and each symbol represents values from individual recipient mice. (**E** and **F**) Graphs depicting the number of single-base-pair mutations (E) and the ratio of replacement: silent mutations (F) in the CDR1 of V_H_186.2 among sorted donor-derived GC B cells in young or aged recipients at day 14 postimmunization. Each symbol represents values from a single sorted GC B cell. Statistics were calculated using unpaired Mann–Whitney *U* test. Data are pooled from two independent repeat experiments. wo, wk old.

B1-8i Tg (19) and WT C57BL/6 mice were bred and maintained in the Babraham Institute Biological Support Unit, where B1-8i BCR-Tg mice were also aged. No primary pathogens or additional agents listed in the FELASA recommendations (20) were detected during health-monitoring surveys of the stock holding rooms. Ambient temperature was ∼19°C–21°C and relative humidity 52%. Lighting was provided on a 12-h light/12-h dark cycle, including 15-min “dawn” and “dusk” periods of subdued lighting. After weaning, mice were transferred to individually ventilated cages with one to five mice per cage. Mice were fed CRM (P) VP diet (Special Diet Services) ad libitum and received seeds (e.g., sunflower, millet) at the time of cage cleaning as part of their environmental enrichment. All mouse experimentation was approved by the Babraham Institute Animal Welfare and Ethical Review Body. Animal husbandry and experimentation complied with existing European Union and U.K. Home Office legislation and local standards (PPL: P4D4AF812). Young adult B1-8i mice were 6–12 wk old, and aged B1-8i mice were at least 90 wk old when used for experiments. Young recipient C57BL/6 mice were 8–12 wk old, and aged recipient C57BL/6 mice were at least 90 wk old at the time of immunization. The donor and recipient mice used in the experiments for Figs. 1–4 were female, whereas the donor and recipient mice used in the experiments for Fig. 5 were male.

### Adoptive transfers of B1-8i cells

Single-cell suspensions of spleen and mesenteric and peripheral lymph nodes (LNs) from young 6- to 12-wk-old adult or >90-wk-old aged B1-8i mice were obtained by pressing the tissues through a 70-µm mesh in PBS with 2% FBS under sterile conditions. B cells were then enriched using the MagniSort Mouse B cell Enrichment Kit (8804-6827-74; Thermo Fisher Scientific), according to the manufacturer’s instruction. Cell numbers and viability were determined using a CASY TT Cell Counter (Roche). A small aliquot of enriched B cells was taken and stained to determine the percentage of NP-binding B cells by flow cytometry before cell transfer. The cell suspensions were then diluted in appropriate volumes of PBS to obtain a final concentration of 1 × 10^5^ NP-binding B cells per milliliter. A total of 100 μl of 1 × 10^4^ NP-binding B cells from young and aged donor B1-8i-Tg mice were injected i.v. into the tail of congenic WT recipients. Recipient mice were then immunized s.c. with NP–keyhole limpet hemocyanin (KLH; NP-KLH)/Alum, as detailed later, and draining inguinal LNs (iLNs) were collected at the indicated time points for flow cytometry.

### s.c. immunizations with NP-KLH/Alum

To induce GCs in iLNs, we immunized recipient C57BL/6 mice s.c. on both flanks on the lower part of the body with NP-KLH/Alum (N-5060-25; Biosearch Technologies). NP-KLH was first diluted in PBS, and the same volume of Imject Alum (77161; Thermo Scientific) was added to reach a final concentration of 0.5 mg/ml NP-KLH. After 30 min of vortexing, 100 μl of emulsion was injected s.c. into the hind flanks of the experimental mice, which have received the i.v. transfer of donor B cells.

### Phenotyping of B1-8i cells using flow cytometry

A small aliquot (1–2 × 10^6^) of enriched B cells from the young adult or aged donor mice was stained for phenotypic analysis before transfer. In brief, cells were stained in 96-well V-bottom plates. Surface Ab staining was performed for 2 h at 4 °C in PBS with 2% FCS, in the presence of 2.4G2 hybridoma (hb-197; ATCC) tissue culture supernatant and Rat IgG isotype control (10700; Invitrogen) to block nonspecific binding via Fc interactions. After incubation, samples were washed twice with PBS with 2% FCS, before they were fixed with the eBioscience Foxp3/Transcription Factor Staining Buffer (00-5323-00) for 30 min at 4°C. The samples were then washed twice with 1× permeabilization buffer (00-8333-56; eBioscience) and stained with the intracellular Ab mix in permeabilization buffer at 4°C overnight. After overnight incubation, the samples were washed twice with 1× permeabilization buffer and washed once with PBS with 2% FCS, before they were acquired on an Aurora Spectral Cytometer (Cytek). Cells for single-color controls were prepared in the same manner as the fully stained samples. The Abs used for the surface and intracellular staining are listed in Tables I.

**Table I.** List of Abs used for flow cytometric analysis of B1-8i cells pretransfer

Abs Used in Stains	Company and Clone	Identifier	Dilution
Abs used in surface stain			
BUV563-coupled anti-mouse CXCR4	BD (2B11/CXCR4)	741313	1:400
BV480-coupled anti-mouse IgG1	BD (A85-1)	746811	1:500
BV650-coupled anti-mouse CXCR3	BioLegend (CXCR3-173)	126531	1:500
BV785-coupled anti-mouse CXCR5	BioLegend (L138D7)	145523	1:200
PE-coupled NP	Biosearch Technologies	N-5070-1	1:100
PE-Cy7-coupled anti-mouse CD40	BioLegend (3/23)	124622	1:2000
A700-coupled anti-mouse MHCII IA/IE	Invitrogen (M5/114.15.2)	56-5321-82	1:2000
ViaKrome808-coupled Live/Dead	Beckman Coulter	C36628	1:2000
APC-Fire750-coupled anti-mouse CD86	BioLegend (GL-1)	105045	1:1000
Abs used in intracellular stain			
BUV395-coupled anti-mouse CD11c	BD (N418)	744180	1:1000
BUV496-coupled anti-mouse CD23	BD (B3B4)	741058	1:2000
BUV661-coupled anti-mouse CD19	BD (1D3)	612971	1:2000
BUV737-coupled anti-mouse CD62L	BD (MEL-14)	612833	1:1000
Pacific Blue-coupled IRF4	BioLegend (IRF4.3E4)	646418	1:1000
BV510-coupled anti-mouse CD21	BD (7G6)	747764	1:2000
AF488-coupled anti-mouse GL7	Invitrogen (GL-7)	53-5902-82	1:1000
Spark YG593-coupled anti-mouse CD11b	BioLegend (M1/70)	101282	1:2000
PE-Fire 640-coupled anti-mouse CD38	BioLegend (90)	102744	1:2000
Spark NIR685-coupled anti-mouse IgD	BioLegend (11-26c.2a)	405750	1:2000
APCFire810-coupled anti-mouse B220	BioLegend (RA3-6B2)	103278	1:1000

AF, Alexa Fluor; APC, allophycocyanin.

### Flow-cytometric analysis of iLNs postimmunization

Cell suspensions of the dissected iLNs from the recipient mice were obtained by pressing the tissues through a 70-µm mesh in PBS with 2% FCS. Cell numbers and viability were determined using a CASY TT Cell Counter (Roche). Cells were washed and transferred into 96-well V-bottom plates. Surface Ab staining was performed for 2 h at 4°C in PBS with 2% FCS, in the presence of 2.4G2 hybridoma (hb-197; ATCC) tissue culture supernatant and Rat IgG isotype control (10700; Invitrogen) to block nonspecific binding via Fc interactions. After incubation, samples were washed twice with PBS with 2% FCS and acquired on an Aurora Spectral Cytometer (Cytek). Cells for single-color controls were prepared in the same manner as the fully stained samples. Flow cytometry data were analyzed using FlowJo v10 software (Tree Star). The Abs used are listed in Table II.

**Table II.** List of Abs used for flow cytometric analysis of mouse LN cells postimmunization

Abs	Company and Clone	Identifier	Dilution
BUV661-coupled anti-mouse CD19	BD (1D3)	565076	1:2000
BUV737-coupled anti-mouse CD4	BD (RM4-5)	612843	1:500
BV480-coupled anti-mouse IgG1	BD (A85-1)	746811	1:500
BV510-coupled anti-mouse CD95	BD (Jo2)	563646	1:200
BV605-coupled anti-mouse CD86	BioLegend (GL-1)	105037	1:200
BV711-coupled anti-mouse CD138	BioLegend (281-2)	142519	1:500
Alexa Fluor 532–coupled IgM	Conjugated in-house (mAb from Thermo Fisher Scientific, clone II/41)		1:500
PerCPCy5.5-coupled anti-mouse CD38	BioLegend (90)	102722	1:400
PE-coupled NP	Biosearch Technologies	N-5070-1	1:100
PE eFluor610-coupled anti-mouse CD45.1	Invitrogen (A20)	61-0453-82	1:200
PE-Cy7-coupled anti-mouse GL-7	BioLegend (GL-7)	144620	1:200
APC-coupled CXCR4	BioLegend (L276F12)	146508	1:400
Spark NIR685-coupled anti-mouse IgD	BioLegend (11-26c.2a)	405750	1:1000
APCFire810-coupled anti-mouse B220	BioLegend (RA3-6B2)	103278	1:500
A700-coupled anti-mouse CD45.2	BioLegend (104)	109822	1:400
APC-e780-coupled Live/Dead	eBioscience	65-0865-14	1:10,000

APC, allophycocyanin.

### VH186.2 PCR and sequencing of single-cell-sorted GC B cells

Cell suspensions from iLNs were obtained as described earlier and stained with the Abs listed in Table III for 2 h at 4°C in PBS with 2% FCS, in the presence of 2.4G2 hybridoma (hb-197; ATCC) tissue culture supernatant and Rat IgG isotype control (10700; Invitrogen) to block nonspecific binding via Fc interactions. Anti-mouse CD3, CD4, CD11c, and Ly6c Abs were included in the dump APC channel to gate out non-B cells. After incubation, samples were washed twice with PBS with 2% FCS, filtered, and resuspended.

**Table III.** List of Abs used for FACS of mouse cells

Abs	Company and Clone	Identifier	Dilution
BUV661-coupled anti-mouse CD19	BD (1D3)	565076	1:500
BV510-coupled anti-mouse CD45.2	BioLegend (104)	109838	1:500
BV605-coupled anti-mouse IgG1	BD (A85-1)	563285	1:500
BV785-coupled anti-mouse B220	BioLegend (RA3-6B2)	103246	1:500
PerCPCy5.5-coupled anti-mouse CD38	BioLegend (90)	102722	1:500
PE-coupled NP	Biosearch Technologies	N-5070-1	1:100
PE-Cy7-coupled anti-mouse GL-7	BioLegend (GL-7)	144620	1:500
APC-coupled anti-mouse CD3	BioLegend (17A2)	100236	1:500
APC-coupled anti-mouse CD4	eBioscience (GK1.5)	17-0041-83	1:500
APC-coupled anti-mouse CD11c	BioLegend (N418)	117310	1:500
APC-coupled anti-mouse Ly6c	Invitrogen (HK1.4)	17-5932-80	1:500
A700-coupled anti-mouse CD45.1	BioLegend (A20)	110724	1:500
APC-e780-coupled Live/Dead	eBioscience	65-0865-14	1:10,000

APC, allophycocyanin.

This method was adapted from Natt and Espéli (21). CD45.1^+^CD45.2^−^NP^+^IgG1^+^B220^+^CD19^+^CD38^−^GL7^+^ cells were single cell sorted into 96-well plates, containing 10 μl of reverse transcription lysis buffer per well (2 U/μl RNase inhibitor (EO0381; Thermo Fisher Scientific), 4 mM DTT (43816; Sigma), 30 ng/μl Random Hexamers (SO142; Thermo Fisher Scientific), 1% Nonidet P-40, 0.2× PBS), using the FACSAria Fusion sorter (BD Biosciences). Plates containing the sorted cells were then stored at −80°C. For reverse transcription, plates were placed in a thermocycler (Bio-Rad), where it is heated to 65°C for 2 min and cooled to 10°C for 5 min. Fifteen microliters of reverse transcription buffer containing 1 mM dNTPs (R0194; Thermo Fisher Scientific), 8 mM DTT (43816; Sigma), 0.2 U/μl RNase inhibitor (EO0381; Thermo Fisher Scientific), and 160 U GoScript Reverse Transcriptase (A5004; Promega) was then added to each well. The thermocycling program is then continued, with the following settings: 22°C for 10 min, 37°C for 30 min, and 90°C for 6 min. The prepared cDNA was stored at −20°C.

For nested PCR, 2.5 μl of the prepared cDNA was used. The PCR mix for the first round of PCR was made up using 10× PCR buffer, dNTP, and Taq DNA polymerase from the HotStar Taq DNA polymerase kit (203205; QIAGEN) with 20 pmol of the following primers: forward 5′-GCTGTATCATGCTCTTCTTG-3′ and reverse 5′-GGATGACTCATCCCAGGGTCACCATGGAGT-3′. Plates were placed in a thermocycler (Bio-Rad) with the following program: 95°C for 15 min, 94°C for 3 min, 39 cycles of 94°C for 45 s, 50°C for 1 min, 72°C for 1 min, and finally 72°C for 10 min. The PCR product was then diluted 30 times in nuclease-free water. One microliter of the first PCR product was then used in the second round of PCR, which was prepared with the HotStar Taq DNA polymerase kit (203205; QIAGEN) and 20 pmol of the following primers: forward 5′-GGTGTCCACTCCCAGGTCCA-3′ and reverse 5′-CCAGGGGCCAGTGGATAGAC-3′. The thermocycling program used for the second PCR was the same as the first. Five microliters of the second PCR product was used to verify positive clones on a 1% agarose gel. The PCR products containing positive clones were purified using the ExoSAP-IT PCR Product Cleanup Reagent (78201; Applied Biosystems), and purified samples were sent for Sanger sequencing to Source Bioscience, U.K. Analysis was performed using an automated alignment pipeline in Perl that aligned sequences to the V_H_186.2 consensus sequence to identify the 33rd codon for each sample, as well as the quantity of replacement and silent mutations for each sequence. Per-sample calls were exported as a .csv for downstream analysis in the Prism v9 software (GraphPad).

### Statistical analysis

All experiments were performed at least twice (three to seven recipient mice per group). Statistical analysis was performed using the Prism v9 and v10 software (GraphPad). Differences between experimental groups were determined using paired Wilcoxon matched pairs signed rank test, unpaired Mann–Whitney *U* test, or two-way ANOVA with Sidak’s multiple comparisons test, where appropriate. The *p* values were considered significant when <0.05.

## Results

### B cells in aged B1-8i Tg mice are less frequently of a follicular phenotype

The B1-8i adoptive transfer system was used in this study to investigate whether there are any cell-intrinsic defects in the ability of B cells from aged mice to undergo affinity maturation. About 10% of B cells from B1-8i Tg mice contain the knock-in canonical B1-8 H chain (V_H_186.2, DFL16.1, and J_H_2) that, when combined with an Igλ L chain, produces an Ab with intermediate affinity for the hapten NP (22). To check for phenotypic differences that might affect B cell responses to immunization, we first stained NP-specific B cells from young adult (6- to 12-wk-old) and aged (>90-wk-old) B1-8i mice with a comprehensive flow cytometry panel (Tables I) to determine their Ig isotype and basal expression levels of markers before transfer. Aged B1-8i mice had on average 10% fewer IgD^+^ naive B cells and follicular B cells (CD23^hi^CD21^int^) among NP-specific B cells, compared with young adult mice (Fig. 1A, 1B). Correspondingly, there was a tendency for aged mice to have higher proportions of marginal zone B cells (CD23^−^CD21^+^), CD23^−^CD21^−^ cells, and CD11c^+^ atypical B cells among the NP-specific B cells (Fig. 1A, 1B). These age-related effects on B1-8i Tg B cell subsets were also observed in the total B cell population (Supplemental Fig. 1). We also assessed the expression of various proteins on the NP-binding B cells: chemokine receptors and trafficking molecules; CXCR4, CD62L, and CXCR5 (Fig. 1C–E); B cell activation markers, CD38, GL7, and IRF4 (Fig. 1F–H); atypical or age-associated B cell markers, CXCR3, CD11b, and CD11c (23) (Fig. 1I–K); and costimulatory molecules, CD86, CD40, and MHC class II (Fig. 1L–N). Further, when total NP-binding B cells were gated into individual B cell subsets and these markers were analyzed, we likewise observed no significant age-dependent differences in the expression levels of these markers (Supplemental Fig. 2).

### NP-specific B cells from aged mice do not have intrinsic defects in becoming GC B cells

To determine whether there are any cell-intrinsic defects by B cells from aged mice in responding to immunization, we adoptively transferred equal numbers of NP-specific B cells from either a young adult or aged B1-8i Tg mouse into young (8- to 12-wk-old) congenic WT recipient mice. Their responses were assessed in draining iLNs of recipient mice at days 6, 10, 14, 21, and 28 postimmunization with NP-KLH/Alum (Fig. 2A, Tables II). Across all time points, there was no significant difference in the percentage and number of NP-specific B cells derived from the young adult or aged donor mice in recipient iLNs (Fig. 2B). NP-specific B cells from aged mice also had no significant defects in class-switch recombination to IgG1 (Fig. 2C, 2D) or in becoming GC B cells (Fig. 2E, 2F). There was also no significant difference in the percentage and number of short-lived extrafollicular plasma cells derived from donor cells from aged mice, compared with those from the young adult mice (Fig. 2G, 2H). Together, this shows that there are no cell-intrinsic defects in the ability of NP-specific B cells from aged B1-8i Tg mice in responding to stimulation and differentiating into plasma cells or GC B cells.

### NP-specific B cells from aged mice receive positive selection signals in the light zone

Positive selection is a key process that primarily occurs in the light zone (LZ) of the GC, and that is essential for affinity maturation. Using the aforementioned adoptive transfer system (Fig. 2A), we assessed the response of transferred B1-8i B cells, which carry a cMyc-GFP reporter gene, at the peak of the GC response, day 6 postimmunization (Fig. 2E, 2F; gating strategy is shown in Fig. 3A). The cMyc^+^ GC B cells identified were predominantly of the LZ phenotype (CD86^hi^CXCR4^+^ GC B cells) (4) (Fig. 3B). NP-specific GC B cells and LZ GC B cells derived from aged mice showed no intrinsic defects in cMyc upregulation (Fig. 3C, 3D), suggesting that age does not diminish the ability of B cells to receive positive selection signals in a young microenvironment. This suggests that this mechanism that underpins affinity maturation is intact in B cells with age.

### NP-specific B cells from aged mice undergo mutation and selection of high-affinity clones

The ability of B cells from aged mice to undergo somatic hypermutation and acquire high-affinity mutations was investigated by sequencing the V_H_186.2 region of sorted donor-derived NP-specific GC B cells (Tables III) from the iLNs of young adult recipient mice at day 14 postimmunization. Substitution of a tryptophan (W) with a leucine (L) at position 33 of the CDR1 of V_H_186.2 results in a 10-fold increase in Ab affinity for NP (24). No significant difference was shown in the frequency of W33L mutation among sorted NP-specific IgG1^+^ GC B cells from young adult and aged donor mice (Fig. 4A, 4B). In addition, the frequency of mutations and the ratio of replacement to silent mutations were similar among GC B cells from young adult and aged mice (Fig. 4C, 4D). Together, this suggests that B cells from aged mice are equally functional in undergoing somatic hypermutation and receiving positive selection signals, ultimately producing GC B cell clones with affinity-enhancing mutations in a young microenvironment.

### NP-specific B cells from young mice have fewer mutations and high-affinity clones in GCs when transferred into aged mice

Because B cells from aged mice were capable of undergoing affinity maturation, we hypothesized that B cell–extrinsic factors contribute to the age-related impairments observed after immunization. NP-specific B cells from young adult B1-8i Tg mice were adoptively transferred into young (8- to 12-wk-old) or aged (>90-wk-old) congenic WT recipient mice, and the B cell response was analyzed at day 14 postimmunization with NP-KLH in Alum (Fig. 5A). NP-binding B cells were able to participate in the GC response in both adult and aged recipient mice (Fig. 5B), enabling the assessment of somatic hypermutation and acquisition of high-affinity variants by sequencing. Sorted NP-specific IgG1^+^ GC B cells from young adult donor mice showed significant defects in their ability to acquire the affinity-enhancing W33L mutation when transferred into aged recipient mice, compared with those transferred into young mice (Fig. 5C, 5D). Furthermore, donor-derived GC B cells in aged recipient mice had significantly lower rates of mutation in the V_H_186.2 region (Fig. 5E) and a lower ratio of replacement to silent mutations (Fig. 5F) than those in young recipients. Together, this shows that an aged microenvironment is a key contributor to defects in affinity maturation upon immunization.

## Discussion

In this study, we used an in vivo adoptive transfer system to show that B cells from aged mice did not exhibit cell-intrinsic defects in affinity maturation postimmunization, and that the aged microenvironment has a dominant role in driving age-related impairments. We first showed that aging results in a reduction in the percentage of naive B cells and follicular B cells, and a concomitant increase in the proportions of marginal zone B cells and CD11c^+^ atypical B cells in the LNs of B1-8i Tg mice. These trends are consistent with previous reports in the spleens of aged mice, suggesting global changes in B cell subset proportions with age across organs (23, 25). Follicular B cells are known to dominate T-dependent responses to protein Ags by differentiating into short-lived extrafollicular plasma cells or GC-derived long-lived plasma cells (26). In contrast, marginal zone B cells and CD11c^+^ atypical B cells have been shown to rapidly differentiate into short-lived plasmablasts in a GC-independent manner (27, 28). This might account for the trend toward an increase in plasmablasts derived from B cells from aged mice early postimmunization observed in this study and in previous work (17). Nevertheless, despite a lower proportion of follicular B cells among transferred NP-specific B cells from aged mice, NP-specific B cells from aged mice were not defective in becoming plasma cells or GC B cells, compared with those from young adult mice. B cells from aged mice also had no intrinsic defects in class switching to IgG1^+^ cells postimmunization, consistent with previous work (17, 18, 29). Although we previously observed that B cells from aged SW_HEL_ mice mounted a smaller early GC response compared with those from young adult mice (17), we did not observe the same delayed kinetics in this system, suggesting some differences attributed to the model used. Some factors that might contribute to this discrepancy include differences in Ag used that might result in distinct downstream BCR signaling after stimulation and also differences in the organ analyzed, spleens in the SW_HEL_ model and iLNs in the B1-8i model used in this study. Nevertheless, in both transfer systems, Ag-specific B cells from aged mice did not have intrinsic defects in mounting a peak GC response, implicating B cell–extrinsic factors as causal in age-dependent defects in GC formation and magnitude.

In this study, we also showed that NP-specific GC B cells derived from B cells of aged mice were equally able to upregulate cMyc as those from young adult mice. cMyc expression is induced in GC B cells selected for their favorable BCRs, which allows their cyclic reentry into the dark zone for further proliferation and somatic hypermutation (4, 6). The lack of defects in cMyc upregulation shown in this article indicated that B cells from aged mice are able to interact with and receive positive survival signals from Tfh cells and follicular dendritic cells normally. By contrast, it has been shown that when B1-8i B cells from young adult mice are transferred into aged recipients, cMyc upregulation is impaired (14). This is consistent with the data shown in this article that it is the aged microenvironment, rather than the cell-intrinsic changes with age in B cells, that is responsible for altered positive selection in the GC.

One limitation to our study is that the model system used, involving B cells with Tg BCRs, is unable to account for B cell–intrinsic changes in BCR repertoire, which has been shown to contract with age (30, 31). Reconstitution of B10 SCID mice with polyclonal B cells from aged mice was previously shown to result in lower mutation frequencies in the V_H_ sequences, compared with reconstitution with B cells from young mice (13). This might hint at a B cell–intrinsic defect in somatic hypermutation with age, although the number of sequences analyzed in the study (13) was considerably lower (a total of 17–40 sequences analyzed per group) than what was analyzed in this study. As such, whether age-related changes in the BCR repertoire affect the ability of B cells to undergo affinity maturation remains to be characterized. Another limitation to our study is that the B cell response in this study is driven by the NP hapten, and thus the response of aged B cells to complex proteins remains unclear. A previous study on influenza virus vaccination has shown that activated B cells from older people tend to target highly conserved but less potent epitopes compared with those from younger people in response to drifted strains of influenza virus, because of reduced rates of de novo somatic hypermutation (11). Whether this defect in adaptability in response to complex immunogens such as influenza virus is driven by B cell–intrinsic changes with age remains to be understood.

Using the same B1-8i adoptive transfer model, we also showed that NP-specific GC B cells from young donor mice had reduced rates of somatic hypermutation, and lower frequencies of replacement mutations and the affinity-enhancing W33L mutations upon transfer into aged mice, suggesting impairments in the ability of young B cells to undergo affinity maturation in an aged environment. This is consistent with a previous report of NP-specific GC B cells showing reduced cMyc expression in aged recipient mice after immunization (14). Together, this suggests that an aged microenvironment results in defects in the ability of B cells to undergo processes of selection and somatic hypermutation, to ultimately produce high-affinity clones during immunization. Some contributing factors in the aged microenvironment include defects in Tfh cell help in the GC and changes in follicular dendritic cells in aging (14, 32).

Our results collectively reveal that B cells from aged mice have no intrinsic defects in going through the cellular process that underpins affinity maturation in the GC after immunization, when the BCR is fixed. This implicates B cell–extrinsic factors as the key contributors to defects in the GC response and humoral immunity with age. Vaccine strategies aimed at improving vaccine responses in older people should therefore be targeted toward improving the aged microenvironment, for example, by rejuvenating Tfh differentiation and boosting stromal cell responses, to promote optimal B cell responses (32, 33, 34).

## Supplementary Material

Supplemental 1 (PDF)
