# B cells from aged mice do not have intrinsic defects in affinity maturation in response to immunisation

**DOI:** 10.4049/jimmunol.2300318

**Published:** 2023-11-15

**Authors:** Jia Le Lee, Silvia Innocentin, Alyssa Silva-Cayetano, Stephane M. Guillaume, Michelle A. Linterman

**Affiliations:** 1 Immunology Program, Babraham Institute, Babraham Research Campus, Cambridge CB22 3AT, UK

## Abstract

Affinity maturation, the progressive increase in serum antibody affinity after vaccination, is an essential process that contributes to an effective humoral response against vaccines and infections. Germinal centres (GCs) are key for affinity maturation, as they are where B cells undergo somatic hypermutation of their immunoglobulin genes in the dark zone, before going through positive selection in the light zone via interactions with T follicular helper cells and follicular dendritic cells. In aged mice, affinity maturation has been shown to be impaired after immunisation, but whether B cell-intrinsic factors contribute to this defect remains unclear. In this study, we show that B cells from aged B cell receptor transgenic mice are able to become GC B cells, which are capable of receiving positive selection signals to a similar extent as B cells from young adult mice. Consistent with this, ageing also does not impact the ability of B cells to undergo somatic hypermutation and acquire affinity-enhancing mutations. By contrast, transfer of B cells from young adult B cell receptor mice into aged recipients resulted in the impaired acquisition of affinity-enhancing mutations, demonstrating that the aged microenvironment causes altered affinity maturation.

## Introduction

Affinity maturation, the progressive increase in the affinity of serum antibodies over time, is an important process that underlies an effective humoral response against vaccines and infections (1, 2). Germinal centres (GCs) are the cellular engines of affinity maturation. Within GCs, B cells undergo somatic hypermutation of their immunoglobulin genes in the dark zone, which generates a pool of B cells carrying random mutations that will then undergo selection in the light zone (3). B cells carrying functional B cell receptors (BCRs) that can uptake and present antigen to T follicular helper (Tfh) cells will receive positive selection signals, which induces upregulation of the proto-oncogene cMyc and promotes cyclic re-entry in the dark zone for further somatic mutations and clonal expansion (4–6). Eventually, these B cells exit the GC as memory B cells or long-lived antibody-secreting plasma cells, which are key in conferring protection against future infections.

The impaired vaccine response during ageing has been widely characterized across different vaccine formulations. This defect involves not only a quantitative reduction in vaccine-specific antibody titres (7–9), but also higher incidence of non-specific autoantibodies (10), and reduced adaptability of B cell response to drifted virus strains in older individuals (11). Analysis of the immunoglobulin genes of GC B cells from the Peyer's patches and spleens of older people revealed that the mechanism of somatic hypermutation is unaltered with age (12). However, *in vivo* studies in mice show that affinity maturation is impaired with age in response to immunisation, as shown by fewer high-affinity GC B cells and fewer antigen-specific plasma cells in the bone marrow of aged mice post-vaccination (13–15). This age-related defect in GC response and output is a result of a reduction in GC magnitude as well as impairment in the selection process (13–16).

Since an effective humoral response during vaccination relies on the coordinated interaction of multiple cell types, a multitude of factors can contribute to age-related defects in vaccine responses. Previous studies have shown that B cells from older people and aged mice have no intrinsic defects in responding to stimulation and differentiating into plasma cells (13, 17, 18). However, whether there are B cell-intrinsic defects in the process of affinity maturation remains unclear. In this study, we tracked the response over time of antigen-specific B cells derived from 6-12 weeks old young adult and >90 weeks old aged B1-8i B cell receptor transgenic mice transferred into young recipient mice, following immunisation. B cells from aged mice had no defects in undergoing class-switch recombination, nor in becoming GC B cells or plasmablasts, compared to those from young adult mice. We also show that B cells derived from aged B1-8i mice were equally able to upregulate cMyc in the GC, suggesting no intrinsic defects in their ability to receive positive selection signals. Sequencing of the V_H_186.2 heavy chain region of NP-specific GC B cells derived from young and aged B1-8i mice revealed no age-related intrinsic defects in the rate of somatic hypermutation nor their ability to acquire the affinity-enhancing W33L mutation. Conversely, NP-specific GC B cells from young donor B1-8i mice displayed defects in somatic mutation frequency and the acquisition of the W33L mutation when transferred into aged recipient mice. This highlights that the poor quality of the GC response in ageing is not due to B cell-intrinsic impairments. Rather, B cell-extrinsic factors are bigger contributors to age-related impairments in affinity maturation in response to immunisation.

## Materials and Methods

### Mouse Husbandry and Maintenance

**Figure 1:**
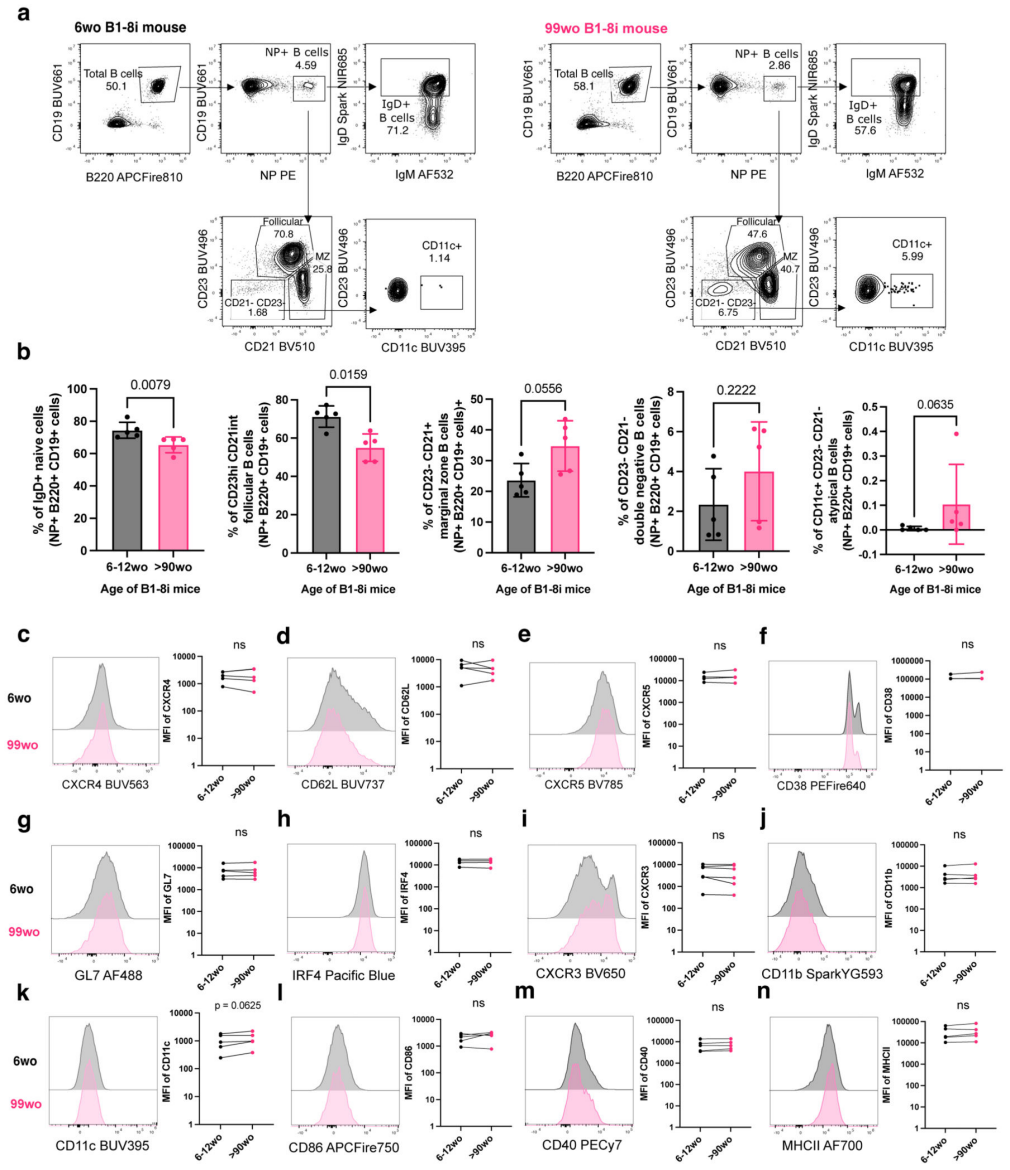
NP-specific B cells from aged mice are phenotypically similar to those from young adult mice. **(a)** Gating strategy for NP-specific IgD+ B cells, follicular B cells (CD23hi CD21int), marginal zone B cells (CD23- CD21+), CD23- CD21- cells, and atypical B cells (CD23- CD21- CD11c+) in young (6wo) and aged (99wo) B1-8i transgenic mice. Numbers adjacent to gates indicate percentage of parent population. Cells were pre-gated for live cells and single cells. **(b)** Graphs depicting the percentage of IgD+ cells, follicular cells (CD23hi CD21int), marginal zone cells (CD23- CD21+), CD23- CD21- cells, and CD11c+ CD23- CD21- cells among NP+ B220+ CD19+ cells in young (6-12wo) and aged (90-100wo) B1-8i transgenic mice. Bar height corresponds to the mean, error bars indicate standard deviation, and each symbol represents values from individual mice. Statistics were calculated using unpaired Mann-Whitney U test. Data pooled from five independent repeat experiments. **(c-n)** Representative flow cytometric histograms and graphs showing the mean fluorescence intensities (MFI) of CXCR4 **(c)**, CD62L **(d)**, CXCR5 **(e)**, CD38 **(f)**, GL7 **(g)**, IRF4 **(h)**, CXCR3**(i)**, CD11b (j), CD11c **(k)**, CD86 **(l)**, CD40 **(m)**, and MHCII **(n)** of NP+ B220+ CD19+ cells from young (6wo) or aged (99wo) B1-8i transgenic mice. Each symbol represents values from individual mice, with young and aged donors from the same experiment shown as paired values. Statistics were calculated using Wilcoxon matched pairs signed rank test. Data pooled from at least four independent repeat experiments.

B1-8i BCR-Tg (19) and WT C57BL/6 mice were bred and maintained in the Babraham Institute Biological Support Unit (BSU), where B1-8i Tg mice were also aged. No primary pathogens or additional agents listed in the FELASA recommendations (20) were detected during health monitoring surveys of the stock holding rooms. Ambient temperature was ~19-21°C and relative humidity 52%. Lighting was provided on a 12 hr light: 12 hr dark cycle including 15 min ‘dawn’ and ‘dusk’ periods of subdued lighting. After weaning, mice were transferred to individually ventilated cages with 1–5 mice per cage. Mice were fed CRM (P) VP diet (Special Diet Services) ad libitum and received seeds (e.g. sunflower, millet) at the time of cage-cleaning as part of their environmental enrichment. All mouse experimentation was approved by the Babraham Institute Animal Welfare and Ethical Review Body. Animal husbandry and experimentation complied with existing European Union and United Kingdom Home Office legislation and local standards (PPL: P4D4AF812). Young adult B1-8i mice were 6-12 weeks old, and aged B1-8i mice were at least 90 weeks old when used for experiments. Young recipient C57BL/6 mice were 8-12 weeks old, and aged recipient C57BL/6 mice were at least 90 weeks old at the time of immunisation. The donor and recipient mice used in the experiments for Figures 1 [Fig F2] [Fig F3] to 4 were female while the donor and recipient mice used in the experiments for Figure 5 were male.

### Adoptive transfers of B1-8i cells

Single cell suspensions of spleen and mesenteric and peripheral lymph nodes from young 6-12 weeks old adult or >90 weeks old aged B1-8i mice were obtained by pressing the tissues through a 70 μm mesh in PBS with 2% foetal bovine serum under sterile conditions. B cells were then enriched using the MagniSort Mouse B cell Enrichment Kit (#8804-6827-74 Thermo Fisher Scientific), according to the manufacturer's instruction. Cell numbers and viability were determined using a CASY TT Cell Counter (Roche). A small aliquot of enriched B cells was taken and stained to determine the percentage of NP-binding B cells by flow cytometry before cell transfer. The cell suspensions were then diluted in appropriate volumes of PBS to obtain a final concentration of 1 x 10^5^ NP-binding B cells/mL. 100μL of 1 x 10^4^ NP-binding B cells from young and aged donor B1-8i-Tg mice were injected intravenously into the tail of congenic WT recipients. Recipient mice were then immunised subcutaneously with NP-KLH/Alum, as detailed below, and draining inguinal LNs (iLNs) were collected at the indicated time points for flow cytometry.

### Subcutaneous immunisations with NP-KLH/Alum

To induce GCs in iLNs, recipient C57BL/6 mice were immunised subcutaneously on both flanks on the lower part of the body with NP-KLH (4-hydroxy-3-nitrophenylacetyl (NP)-Keyhole Limpet Hemocyanin (KLH), #N-5060-25 Biosearch Technologies). NP-KLH was first diluted in PBS and the same volume of Imject™ Alum (ThermoScientific #77161) was added to reach a final concentration of 0.5mg/mL NP- KLH. After 30 min of vortexing, 100μL of emulsion were injected subcutaneously (s.c.) into the hind flanks of the experimental mice, which have received the intravenous transfer of donor B cells.

### Phenotyping of B1-8i cells using Flow Cytometry

A small aliquot (1-2 x 10^6^) of enriched B cells from the young adult or aged donor mice was stained for phenotypic analysis prior to transfer. Briefly, cells were stained in 96-well v-bottom plates. Surface antibody staining was performed for 2 hours at 4 °C in PBS with 2% FCS, in the presence of 2.4G2 hybridoma (ATCC hb-197) tissue culture supernatant and Rat IgG isotype control (Invitrogen, #10700) to block non-specific binding via Fc interactions. Following incubation, samples were washed twice with PBS with 2% FCS, before they were fixed with the eBioscience Foxp3/Transcription Factor Staining Buffer (#00-5323-00) for 30 min at 4°C. The samples were then washed twice with 1 x permeabilisation buffer (eBioscience #00-8333-56) and stained with the intracellular antibody mix in permeabilisation buffer at 4°C overnight. Following overnight incubation, the samples were washed twice with 1 x permeabilisation buffer and washed once with PBS with 2% FCS, before they were acquired on an Aurora Spectral Cytometer (Cytek). Cells for single colour controls were prepared in the same manner as the fully stained samples. The antibodies used for the surface and intracellular staining are listed in Table 1.

### Flow Cytometric Analysis of iLNs post-immunisation

Cell suspensions of the dissected iLNs from the recipient mice were obtained by pressing the tissues through a 70 μm mesh in PBS with 2% FCS. Cell numbers and viability were determined using a CASY TT Cell Counter (Roche). Cells were washed and transferred into 96-well v-bottom plates. Surface antibody staining was performed for 2 hours at 4 °C in PBS with 2% FCS, in the presence of 2.4G2 hybridoma (ATCC hb-197) tissue culture supernatant and Rat IgG isotype control (Invitrogen, #10700) to block non-specific binding via Fc interactions. Following incubation, samples were washed twice with PBS with 2% FCS and acquired on an Aurora Spectral Cytometer (Cytek). Cells for single colour controls were prepared in the same manner as the fully stained samples. Flow cytometry data were analysed using FlowJo v10 software (Tree Star). The antibodies used are listed in Table 2.

### VH186.2 PCR and Sequencing of Single Cell Sorted GC B cells

Cell suspensions from iLNs were obtained as described above, and stained with the antibodies listed in Table 3 for 2 hours at 4 °C in PBS with 2% FCS, in the presence of 2.4G2 hybridoma (ATCC hb-197) tissue culture supernatant and Rat IgG isotype control (Invitrogen, #10700) to block non-specific binding via Fc interactions. Anti-mouse CD3, CD4, CD11c, Ly6c antibodies were included in the DUMP APC channel to gate out non-B cells. After incubation, samples were washed twice with PBS with 2% FCS, filtered and resuspended.

This method was adapted from Natt and Espéli (21). CD45.1+ CD45.2- NP+ IgG1+ B220+ CD19+ CD38- GL7+ cells were single cell sorted into 96-well plates, containing 10μL of reverse transcription lysis buffer per well (2U/μL RNase inhibitor (#EO0381 Thermo Fisher Scientific), 4mM DTT (#43816 Sigma), 30ng/μL Random Hexamers (#SO142 Thermo Fisher Scientific), 1% NP40, 0.2X PBS), using the FACSAria™ Fusion sorter (BD Biosciences). Plates containing the sorted cells were then stored at -80°C. For reverse transcription, plates were placed in a thermocycler (BioRAD), where it is heated to 65 °C for 2min and cooled to 10 °C for 5min. 15μL of reverse transcription buffer containing 1mM dNTPs (#R0194 Thermo Fisher Scientific), 8mM DTT (#43816 Sigma), 0.2U/μL RNase inhibitor (#EO0381 Thermo Fisher Scientific) and 160U GoScript Reverse Transcriptase (Promega #A5004) was then added to each well. The thermocycling program is then continued, with the following settings: 22 °C for 10min, 37 °C for 30min, 90 °C for 6min. The prepared cDNA was stored at -20 °C.

For nested PCR, 2.5μL of the prepared cDNA was used. The PCR mix for the first round of PCR was made up using 10x PCR buffer, dNTP and Taq DNA polymerase from the HotStar Taq DNA polymerase kit QIAGEN(#203205) with 20 pmol of the following primers: forward-GCTGTATCATGCTCTTCTTG and reverse-GGATGACTCATCCCAGGGTCACCATGGAGT. Plates were placed in a thermocycler (BioRAD) with the following program: 95 °C for 15min, 94 °C for 3min, 39 cycles of 94 °C for 45sec, 50 °C for 1min and 72 °C for 1min, and finally 72 °C for 10min. The PCR product was then diluted 30 times in nuclease-free water. 1μL of the first PCR product was then used in the second round of PCR which was prepared with the HotStar Taq DNA polymerase kit (#203205 QIAGEN) and 20 pmol of the following primers: forward-GGTGTCCACTCCCAGGTCCA and reverse-CCAGGGGCCAGTGGATAGAC. The thermocycling program used for the second PCR was the same as the first. 5μL of the second PCR product was used to verify positive clones on a 1% agarose gel. The PCR products containing positive clones were purified using the ExoSAP-IT™ PCR Product Cleanup Reagent (#78201 Applied Biosystems) and purified samples were sent for Sanger sequencing to Source Bioscience, UK. Analysis was performed using an automated alignment pipeline in Perl that aligned sequences to the V_H_186.2 consensus sequence to identify the 33^rd^ codon for each sample as well as the quantity of replacement and silent mutations for each sequence. Per sample calls were exported as a .csv for downstream analysis in the Prism v9 software (GraphPad).

### Statistical Analysis

All experiments were performed twice or more (3-7 recipient mice per group). Statistical analysis was performed using the Prism v9 and v10 software (GraphPad). Differences between experimental groups were determined using paired Wilcoxon matched pairs signed rank test, unpaired Mann-Whitney *U* test or 2-way ANOVA with Sidak’s multiple comparisons test, where appropriate. p values were considered significant when <0.05.

## Results

### B cells in aged mice are less frequently of a follicular phenotype

The B1-8i adoptive transfer system was used in this study to interrogate if there are any cell-intrinsic defects in the ability of B cells from aged mice to undergo affinity maturation. About 10% of B cells from B1-8i mice contain the knock-in canonical B1-8 heavy chain (V_H_186.2, DFL16.1 and J_H_2) that, when combined with an Igλ light chain, produces an antibody with intermediate affinity for the hapten 4-hydroxy-3-nitrophenylacetyl (NP) (22). In order to check for phenotypic differences that might affect B cell responses to immunisation, NP-specific B cells from young adult (6-12 weeks old) and aged (>90 weeks old) B1-8i mice were first stained with a comprehensive flow cytometry panel to determine their Ig isotype and basal expression levels of markers prior to transfer. Aged B1-8i mice had on average 10% fewer IgD+ naïve B cells and follicular B cells (CD23hi CD21int) among NP-specific B cells, compared to young adult mice (Fig. 1a-b). Correspondingly, there was a tendency for aged mice to have higher proportions of marginal zone B cells (CD23- CD21+), CD23-CD21- cells, and CD11c+ atypical B cells among the NP-specific B cells (Fig. 1a-b). These age-related effects on B1-8i transgenic B cell subsets were also observed in the total B cell population (Supplementary Figure 1). We also assessed the expression of various proteins on the NP-binding B cells: chemokine receptors and trafficking molecules, CXCR4, CD62L, and CXCR5 (Fig. 1c-e), B cell activation markers, CD38, GL7, and IRF4 (Fig. 1f-h), atypical or age-associated B cell markers, CXCR3, CD11b, and CD11c (23) (Fig. 1i-k), and costimulatory molecules, CD86, CD40, and MHCII (Fig. 1l-n). Further, when total NP-binding B cells were gated into individual B cell subsets and these markers analysed, we likewise observed no significant age-dependent differences in the expression levels of these markers (Supplementary Figure 2).

### NP-specific B cells from aged mice do not have intrinsic defects in becoming GC B cells

**Figure 2 F2:**
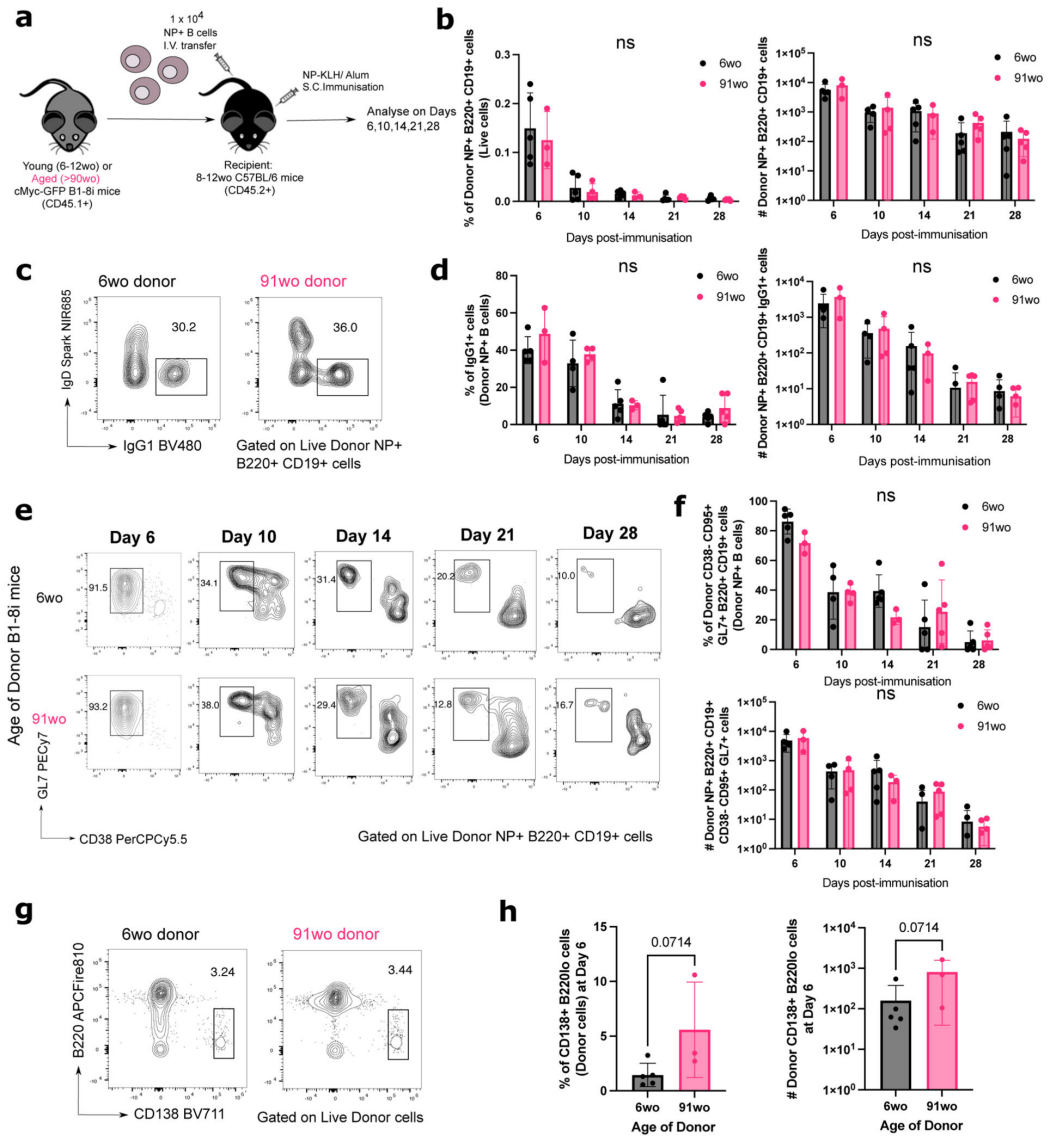
B cells from aged donor mice have no defects in class-switch recombination, entering the GC response and plasmablast differentiation. **(a)** Schematic diagram of adoptive transfer experiments to compare the response of B cells from young (6-12wo) and aged (>90wo) mice in young recipient mice post-immunisation with NP-KLH in alum. Draining inguinal lymph nodes (iLNs) were taken at indicated times post-immunisation for downstream analyses. **(b)** Graphs depicting the percentage and number of donor NP+ B220+ CD19+ cells out of live cells in recipient iLNs at different timepoints post-transfer and immunisation. **(c-f)** Representative flow cytometric plots showing gating strategies for donor-derived IgG1+ IgD- B cells **(c)** and CD38- GL7+ GC B cells **(e)** from 6wo or 91wo donor mice at different timepoints post-transfer and immunisation. Numbers adjacent to gates indicate percentage of donor NP+ B220+ CD19+ cells. Graphs depicting the percentage and number of donor NP+ IgG1+ cells **(d)** and donor NP+ CD38- CD95+ GL7+ cells **(f)** in recipient iLNs at different timepoints post-transfer and immunisation. Statistics were calculated using 2-way ANOVA with Sidak’s multiple comparisons test. **(g)** Representative flow cytometric plots showing gating strategies for donor-derived CD138+ B220lo cells from 6wo or 91wo donor mice at day 6 post-transfer and immunisation. Numbers adjacent to gates indicate percentage of donor cells. **(h)** Graphs depicting the percentage and number of donor CD138+ B220lo cells in recipient iLNs at day 6 post-transfer and immunisation. Bar height corresponds to the mean, error bars indicate standard deviation, and each symbol represents values from individual recipient mice. Statistics were calculated using unpaired Mann-Whitney U test. Data representative of two independent repeat experiments.

To determine if there are any cell-intrinsic defects by B cells from aged mice in responding to immunisation, equal numbers of NP-specific B cells from either a young adult or aged B1-8i mouse were adoptively transferred into young (8-12 weeks old) congenic wild-type recipient mice. Their responses were assessed in draining inguinal lymph nodes (iLNs) of recipient mice at days 6, 10, 14, 21, and 28 post-immunisation with NP conjugated to the Keyhole Limpet Hemocyanin protein (NP-KLH) in Alum (Fig. 2a). Across all timepoints, there was no significant difference in the percentage and number of NP-specific B cells derived from the young adult or aged donor mice in recipient iLNs (Fig. 2b). NP-specific B cells from aged mice also had no significant defects in class-switch recombination to IgG1 (Fig. 2c-d) or in becoming GC B cells (Fig. 2e-f). There was also no significant difference in the percentage and number of short-lived extrafollicular plasma cells derived from donor cells from aged mice, compared to those from the young adult mice (Fig. 2g-h). Together, this shows that there are no cell-intrinsic defects in the ability of NP-specific B cells from aged B1-8i mice in responding to stimulation and differentiating into plasma cells or GC B cells.

### NP-specific B cells from aged mice receive positive selection signals in the light zone

**Figure 3 F3:**
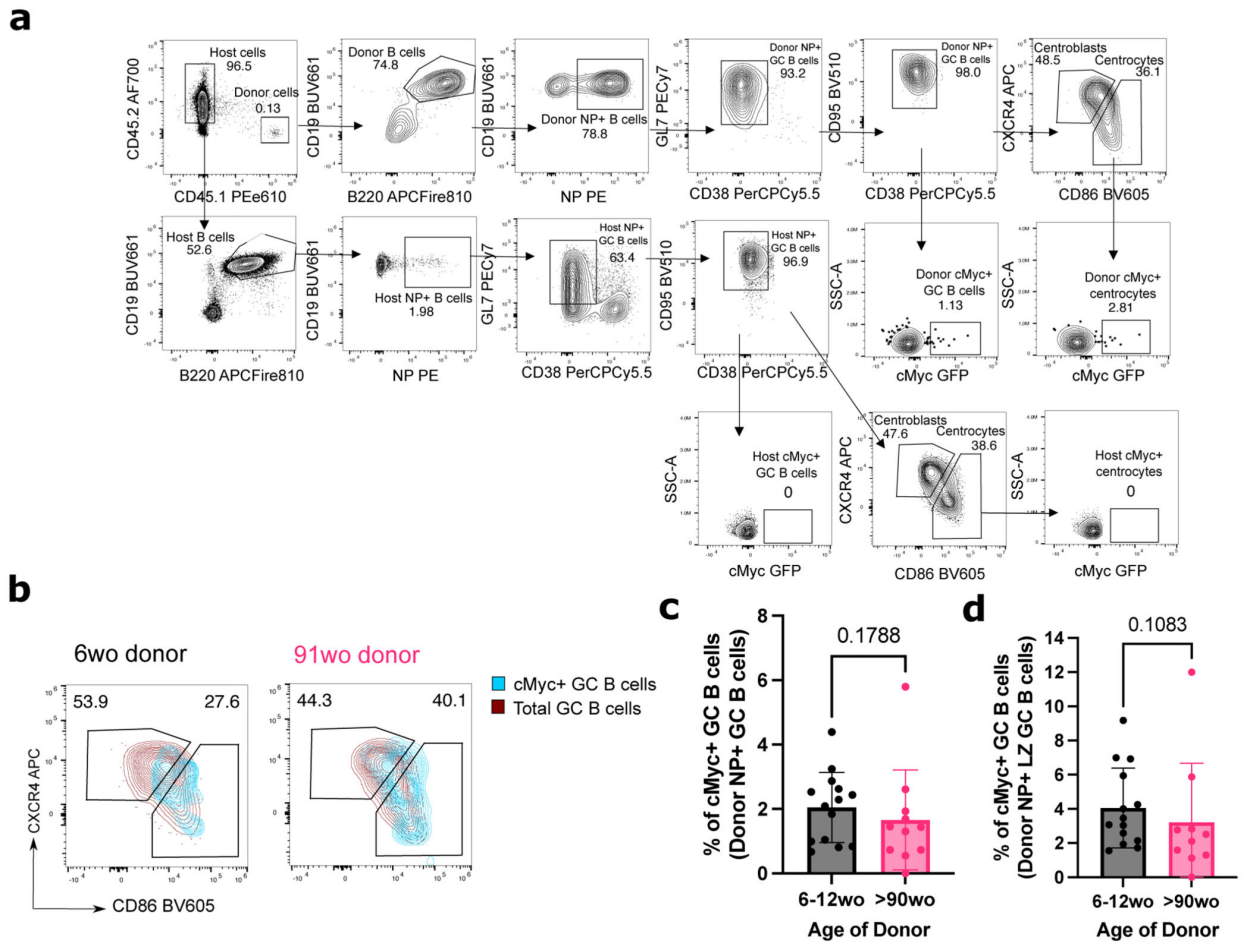
GC B cells from aged donor mice have no intrinsic defects in cMyc upregulation. **(a)** Representative flow cytometric plots showing the gating strategy for cMyc+ cells from donor GC B cells (CD45.1+ CD19+ B220+ NP+ CD38- GL7+ CD95+) and LZ (CXCR4lo CD86hi) GC B cells. **(b)** Flow cytometric plots showing the overlay of cMyc+ GC B cells (blue) on total GC B cells (red) gated for dark zone (CXCR4+CD86lo) and light zone (CXCR4loCD86+) phenotype from young adult or aged B1-8i donor mice. Numbers adjacent to gates indicate percentage of donor NP+ B220+ CD19+ CD38- GL7+ CD95+ GC B cells. **(c-d)** Graphs depicting the percentage of cMyc+ GC B cells out of donor NP+ GC B cells **(c)** and donor NP+ LZ GC B cells **(d)** in recipient iLNs at day 6 post-transfer and immunisation. Bar height corresponds to the mean, error bars indicate standard deviation, and each symbol represents values from individual recipient mice. Statistics were calculated using unpaired Mann-Whitney U test. Data pooled from three independent repeat experiments.

Positive selection is a key process that primarily occurs in the LZ of the GC, and that is essential for affinity maturation. Using the aforementioned adoptive transfer system (Fig. 2a), the response of transferred B1-8i B cells, which carry a cMyc-GFP reporter gene, was assessed at the peak of the GC response, day 6 post-immunisation (Fig. 2e-f) (gating strategy shown in Fig. 3a). The cMyc+ GC B cells identified were predominantly of the LZ phenotype (CD86hi CXCR4+ GC B cells) (4) (Fig. 3b). NP-specific GC B cells and LZ GC B cells derived from aged mice showed no intrinsic defects in cMyc upregulation (Fig. 3c-d), suggesting that age does not diminish the ability of B cells to receive positive selection signals in a young microenvironment. This suggests that this mechanism that underpins affinity maturation is intact in B cells with age.

### NP-specific B cells from aged mice undergo mutation and selection of high affinity clones

**Figure 4:**
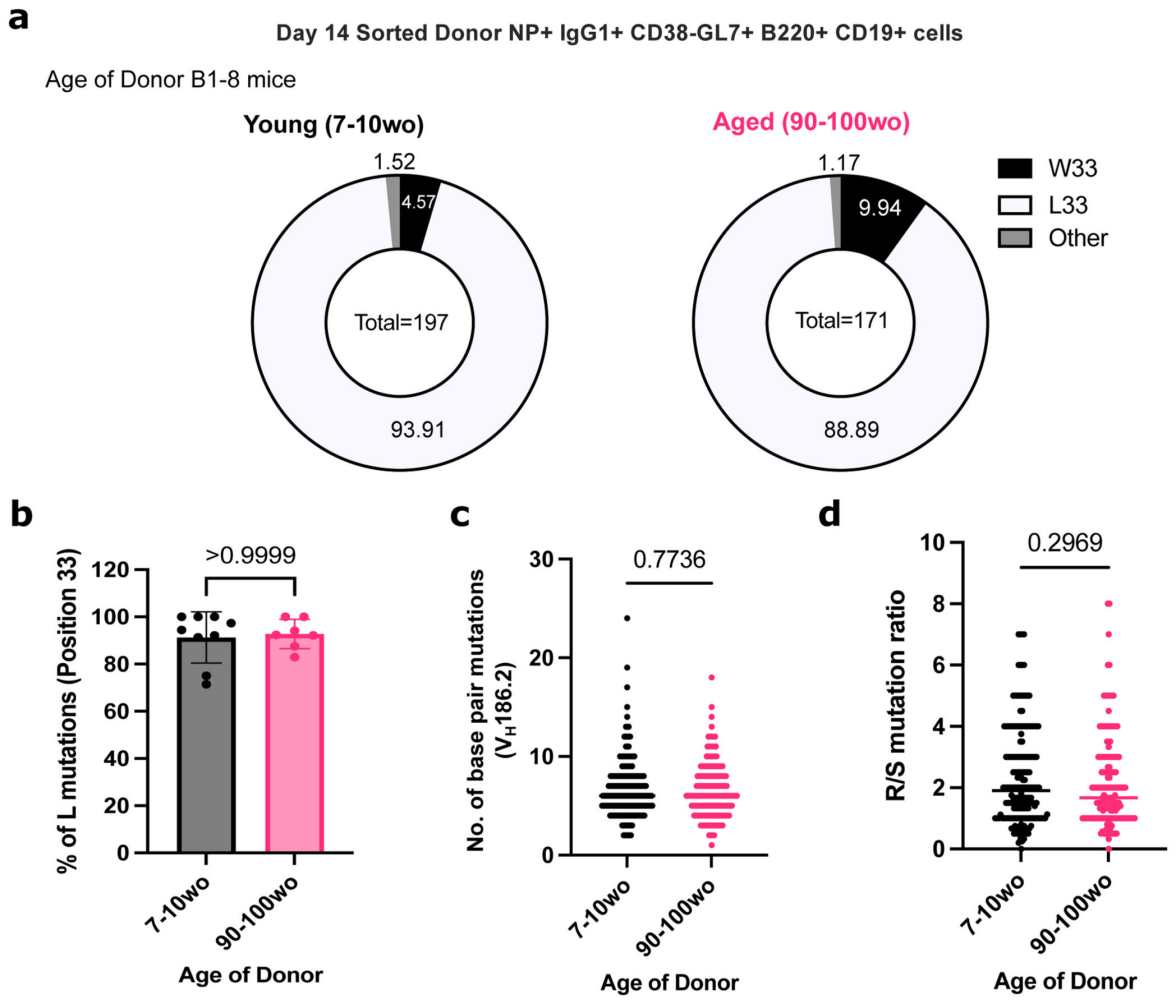
NP-specific B cells from aged mice undergo mutation and selection of high-affinity clones. **(a)** Pie charts indicating the frequency of the affinity-inducing mutation W33L in the CDR1 region of V_H_186.2 sequenced from single cell sorted NP+IgG1+ CD38- GL7+ B220+ CD19+ cells from either young adult (7-10wo) or aged (90-100wo) B1-8i mice in iLNs of young adult (8-12wo) recipient mice 14 days post-immunisation with NP-KLH in Alum. The values in the centre of the pie charts indicate the total number of cells sequenced per group (n=7-9 mice per group from two independent experiments, with an average of 20 GC B cells sequenced per mouse). The number of sequences analysed per recipient mouse is shown in Supplementary Figure 3a. **(b)** Graph depicting the percentage of sorted GC B cells with the W33L mutation from young adult (7-10wo) or aged (90- 100wo) B1-8i mice. Bar height corresponds to the mean, error bars indicate standard deviation, and each symbol represents values from individual recipient mice. **(c-d)** Graphs depicting the number of single base pair mutations **(c)** and the ratio of replacement: silent mutations **(d)** in the CDR1 region of V_H_186.2 among sorted GC B cells derived from young adult (7-10wo) or aged (90-100wo) at day 14 post-immunisation. Each symbol represents values from a single sorted GC B cell from 7-9 recipient mice per group. Statistics were calculated using unpaired Mann-Whitney U test. Data pooled from two independent repeat experiments.

The ability of B cells from aged mice to undergo somatic hypermutation and acquire high-affinity mutations was investigated by sequencing the V_H_186.2 region of sorted donor-derived NP-specific GC B cells from the iLNs of young adult recipient mice at day 14 post-immunisation. Substitution of a tryptophan (W) with a leucine (L) at position 33 of the complementarity-determining region 1 (CDR1) of V_H_186.2 results in a tenfold increase in antibody affinity for NP (24). No significant difference was shown in the frequency of W33L mutation among sorted NP-specific IgG1+ GC B cells from young adult and aged donor mice (Fig. 4a-b). In addition, the frequency of mutations and the ratio of replacement to silent mutations were similar among GC B cells from young adult and aged mice (Fig. 4c-d). Together, this suggests that B cells from aged mice are equally functional in undergoing somatic hypermutation and receiving positive selection signals, and ultimately producing GC B cell clones with affinity-enhancing mutations in a young microenvironment.

### NP-specific B cells from young mice have fewer mutations and high-affinity clones in GCs when transferred into aged mice

**Figure 5:**
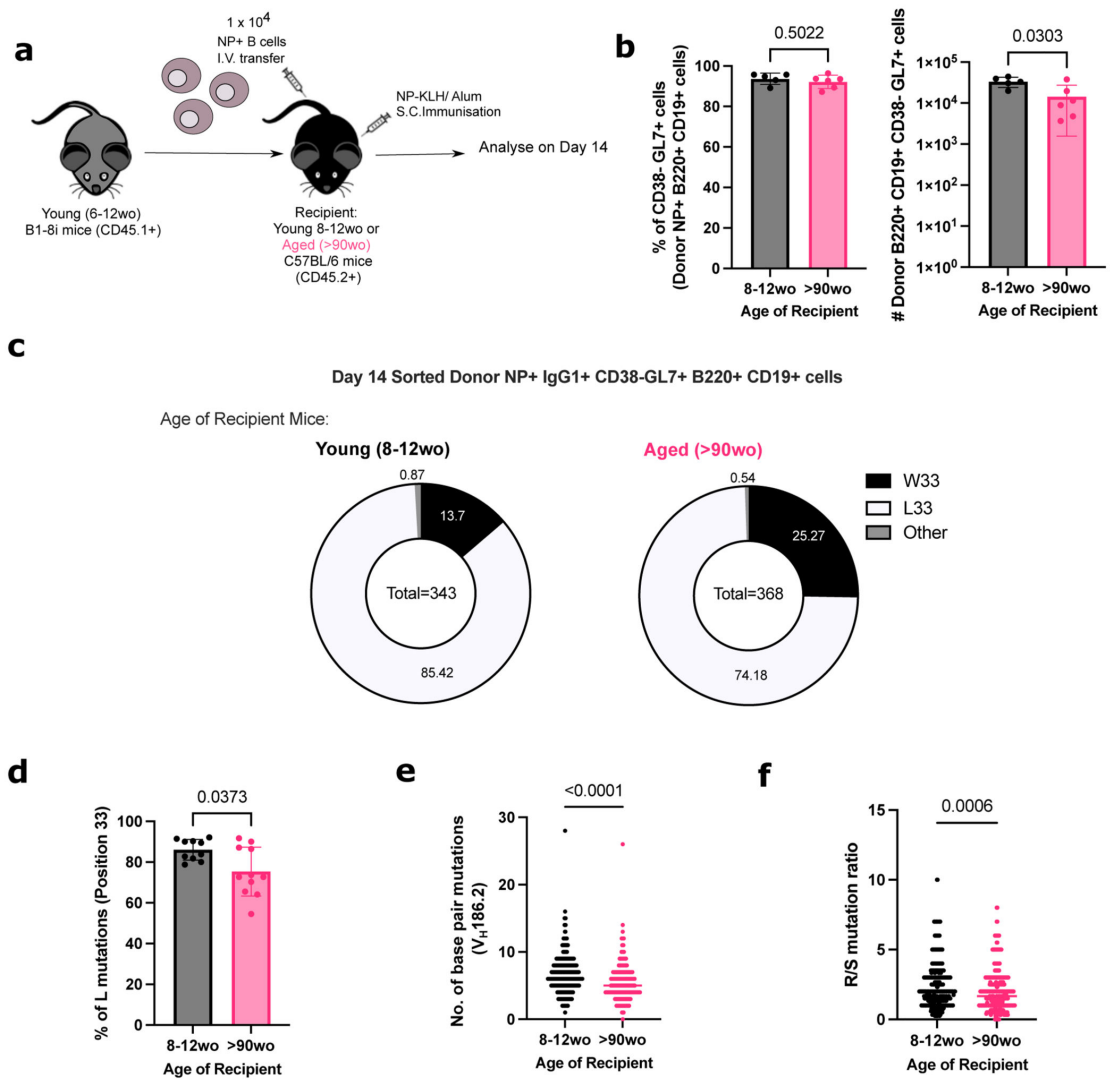
NP-specific B cells from young mice have defects in affinity maturation when transferred into aged recipient mice. **(a)** Schematic diagram of adoptive transfer experiments to compare response of B cells from young (6-12wo) B1-8i mice in young (8-12wo) or aged (>90wo) recipient mice post-immunisation with NP-KLH in alum. Draining inguinal lymph nodes (iLNs) were taken at day 14 post-immunisation for downstream analyses. **(b)** Graphs depicting the percentage and number of donor NP+ CD38- CD95+ GL7+ GC B cells in recipient iLNs at day 14 post-transfer and immunisation. **(c)** Pie charts indicating the frequency of the affinity-inducing mutation W33L in the CDR1 region of V_H_186.2 sequenced from single cell sorted NP+ IgG1+ CD38- GL7+ B220+ CD19+ cells from young adult B1-8i mice in recipient iLNs 14 days post-immunisation. The values in the centre of the pie charts indicate the total number of cells sequenced per group (n=10-11 mice per group from two independent experiments, with an average of 35 GC B cells sequenced per mouse). The number of sequences analysed per recipient mouse is shown in Supplementary Figure 3b. **(d)** Graph depicting the percentage of sorted GC B cells from young adult B1-8i mice with the W33L mutation in young or aged recipients. Bar height corresponds to the mean, error bars indicate standard deviation, and each symbol represents values from individual recipient mice. **(e-f)** Graphs depicting the number of single base pair mutations **(e)** and the ratio of replacement: silent mutations **(f)** in the CDR1 region of V_H_186.2 among sorted donor-derived GC B cells in young or aged recipients at day 14 post-immunisation. Each symbol represents values from a single sorted GC B cell. Statistics were calculated using unpaired Mann-Whitney U test. Data pooled from two independent repeat experiments.

Since B cells from aged mice were capable of undergoing affinity maturation, we hypothesised that B cell-extrinsic factors contribute to the age-related impairments observed after immunisation. NP-specific B cells from young adult B1-8i mice were adoptively transferred into young (8-12 weeks old) or aged (>90 weeks old) congenic wild-type recipient mice, and the B cell response was analysed at day 14 post-immunisation with NP-KLH in Alum (Fig. 5a). NP-binding B cells were able to participate in the GC response in both adult and aged recipient mice (Fig. 5b), enabling the assessment of somatic hypermutation and acquisition of high-affinity variants by sequencing. Sorted NP-specific IgG1+ GC B cells from young adult donor mice showed significant defects in their ability to acquire the affinity-enhancing W33L mutation when transferred into aged recipient mice, compared to those transferred into young mice (Fig. 5c-d). Furthermore, donor-derived GC B cells in aged recipient mice had significantly lower rates of mutation in the V_H_186.2 region (Fig. 5e) and lower ratio of replacement to silent mutations (Fig. 5f) than those in young recipients. Together, this shows that an aged microenvironment is a key contributor to defects in affinity maturation upon immunisation.

## Discussion

In this study, we used an *in vivo* adoptive transfer system to show that B cells from aged mice did not exhibit cell-intrinsic defects in affinity maturation post-immunisation, and that the aged microenvironment has a dominant role in driving age-related impairments. We first showed that ageing results in a reduction in the percentage of naïve B cells and follicular B cells, and a concomitant increase in the proportions of marginal zone B cells and CD11c+ atypical B cells in the lymph nodes of B1-8i mice. These trends are consistent with previous reports in the spleens of aged mice, suggesting global changes in B cell subset proportions with age across organs (23, 25). Follicular B cells are known to dominate T-dependent responses to protein antigens by differentiating into short-lived extrafollicular plasma cells or GC-derived long-lived plasma cells (26). In contrast, marginal zone B cells and CD11c+ atypical B cells have been shown to rapidly differentiate into short-lived plasmablasts in a GC-independent manner (27, 28). This might account for the trend towards an increase in plasmablasts derived from B cells from aged mice early post-immunisation observed in this study and in previous work (17). Nevertheless, despite a lower proportion of follicular B cells among transferred NP-specific B cells from aged mice, NP-specific B cells from aged mice were not defective in becoming plasma cells or GC B cells, compared to those from young adult mice. B cells from aged mice also had no intrinsic defects in class-switching to IgG1+ cells post-immunisation, consistent with previous work (17, 18, 29). While we previously observed that B cells from aged SW_HEL_ mice mounted a smaller early GC response compared to those from young adult mice (17), we did not observe the same delayed kinetics in this system, suggesting some differences attributed to the model used. Some factors that might contribute to this discrepancy include differences in antigen used that might result in distinct downstream BCR signalling following stimulation, and also differences in the organ analysed, spleens in the SW_HEL_ model and iLNs in the B1-8i model used here. Nevertheless, in both transfer systems, antigen-specific B cells from aged mice did not have intrinsic defects in mounting a peak GC response, implicating B cell-extrinsic factors as causal in age-dependent defects in GC formation and magnitude.

Here we also showed that NP-specific GC B cells derived from B cells of aged mice were equally able to upregulate cMyc as those from young adult mice. cMyc expression is induced in GC B cells selected for their favourable BCRs which allows their cyclic reentry into the dark zone for further proliferation and somatic hypermutation (4, 6). The lack of defects in cMyc upregulation shown here indicated that B cells from aged mice are able to interact with and receive positive survival signals from Tfh cells and follicular dendritic cells normally. By contrast, when B1-8i B cells from young adult mice are transferred into aged recipients, cMyc upregulation is impaired (14). This is consistent with the data shown here that it is the aged microenvironment, rather than the cell-intrinsic changes with age in B cells, that is responsible for altered positive selection in the GC.

One limitation to our study is that the model system used, involving B cells with transgenic BCRs, is unable to account for B cell-intrinsic changes in BCR repertoire, which has been shown to contract with age (30, 31). Reconstitution of B10 SCID mice with polyclonal B cells from aged mice was previously shown to result in lower mutation frequencies in the V_H_ sequences, compared to reconstitution with B cells from young mice (13). This might hint at a B cell-intrinsic defect in somatic hypermutation with age, although the number of sequences analysed in the study (13) were considerably lower (a total of 17 to 40 sequences analysed per group) than what was analysed here. As such, whether age-related changes in BCR repertoire affect B cells’ ability to undergo affinity maturation remains to be characterised. Another limitation to our study is that the B cell response here is driven by the NP hapten, and thus the response of aged B cells to complex proteins remains unclear. A previous study on influenza virus vaccination has shown that activated B cells from older people tend to target highly conserved but less potent epitopes compared to those from younger people in response to drifted strains of influenza virus, due to reduced rates of *de novo* somatic hypermutation (11). Whether this defect in adaptability in response to complex immunogens like influenza virus is driven by B cell-intrinsic changes with age remains to be understood.

Using the same B1-8i adoptive transfer model, we also showed that NP-specific GC B cells from young donor mice had reduced rates of somatic hypermutation, and lower frequencies of replacement mutations and the affinity-enhancing W33L mutations upon transfer into aged mice, suggesting impairments in the ability of young B cells to undergo affinity maturation in an aged environment. This is consistent with a previous report of NP-specific GC B cells showing reduced cMyc expression in aged recipient mice following immunisation (14). Together, this suggests that an aged microenvironment results in defects in the ability of B cells to undergo processes of selection and somatic hypermutation, to ultimately produce high-affinity clones during immunisation. Some contributing factors in the aged microenvironment include defects in Tfh cell help in the GC and changes in follicular dendritic cells in ageing (32).

Our results collectively reveal that B cells from aged mice have no intrinsic defects in going through the cellular process that underpins affinity maturation in the GC following immunisation, when the B cell receptor is fixed. This implicates B cell-extrinsic factors as the key contributors to defects in the GC response and humoral immunity with age. Vaccine strategies aimed at improving vaccine responses in older people should therefore be targeted towards improving the aged microenvironment, for example by rejuvenating Tfh differentiation and boosting stromal cell responses, to promote optimal B cell responses (33, 34).

## Supplementary Material

Supplementary figures

## Figures and Tables

**Table 1:** List of antibodies used for flow cytometric analysis of B1-8i cells pre-transfer

	Antibodies used in Surface Stain	Company and Clone	Identifier	Dilution
1	BUV563-coupled anti-mouse CXCR4	BD (2B11/CXCR4)	741313	1:400
2	BV480-coupled anti-mouse IgG1	BD (A85-1)	746811	1:500
3	BV785-coupled anti-mouse CXCR5	BioLegend (L138D7)	145523	1:200
4	PE-coupled NP	Biosearch Technologies	N-5070-1	1:100
5	PE-Cy7-coupled anti-mouse CD40	BioLegend (3/23)	124622	1:2000
6	A700-coupled anti-mouse MHCII IA/IE	Invitrogen (M5/114.15.2)	56-5321-82	1:2000
7	ViaKrome808-coupled Live/Dead	Beckman Coulter	C36628	1:2000
8	APC-Fire750-coupled anti-mouse CD86	BioLegend (GL-1)	105045	1:1000
	**Antibodies used in Intracellular Stain**	**Company and Clone**	**Identifier**	**Dilution**
9	BUV395-coupled anti-mouse CD11c	BD (N418)	744180	1:1000
10	BUV496-coupled anti-mouse CD23	BD (B3B4)	741058	1:2000
11	BUV661-coupled anti-mouse CD19	BD (1D3)	612971	1:2000
12	BUV737-coupled anti-mouse CD62L	BD (MEL-14)	612833	1:1000
13	Pacific Blue-coupled IRF4	BioLegend (IRF4.3E4)	646418	1:1000
14	BV510-coupled anti-mouse CD21	BD (7G6)	747764	1:2000
15	AF488-coupled anti-mouse GL7	Invitrogen (GL-7)	53-5902-82	1:1000
16	Spark YG593-coupled anti-mouse CD11b	BioLegend (M1/70)	101282	1:2000
17	Alexa Fluor 594-coupled anti-mouse Tbet	BioLegend (4B10)	644833	1:500
18	PE-Fire 640-coupled anti-mouse CD38	BioLegend (90)	102744	1:2000
19	Spark NIR685-coupled anti-mouse IgD	BioLegend (11-26c.2a)	405750	1:2000
20	APCFire810-coupled anti-mouse B220	BioLegend (RA3-6B2)	103278	1:1000

**Table 2:** List of antibodies used for flow cytometric analysis of mouse LN cells post-immunisation

	Antibodies	Company and Clone	Identifier	Dilution
1	BUV661-coupled anti-mouse CD19	BD (1D3)	565076	1:2000
2	BUV737-coupled anti-mouse CD4	BD (RM4-5)	612843	1:500
3	BUV615-coupled anti-mouse PD1	BD (RMP1-30)	752354	1:500
4	BV421-coupled anti-mouse CXCR5	BioLegend (L138D7)	145512	1:200
5	BV480-coupled anti-mouse IgG1	BD (A85-1)	746811	1:500
6	BV510-coupled anti-mouse CD95	BD (Jo2)	563646	1:200
7	BV605-coupled anti-mouse CD86	BioLegend (GL-1)	105037	1:200
8	BV711-coupled anti-mouse CD138	BioLegend (281-2)	142519	1:500
9	Alexa Fluor 532-coupled IgM	Conjugated in house (Monoclonal antibody from Thermo Fisher Scientific, clone II/41)		1:500
10	PerCPCy5.5-coupled anti-mouse CD38	BioLegend (90)	102722	1:400
11	PE-coupled NP	Biosearch Technologies	N-5070-1	1:100
12	PE eFluor610-coupled anti-mouse CD45.1	Invitrogen (A20)	61-0453-82	1:200
13	PE-Cy7-coupled anti-mouse GL-7	BioLegend (GL-7)	144620	1:200
14	APC-coupled CXCR4	BioLegend (L276F12)	146508	1:400
15	Spark NIR685-coupled anti-mouse IgD	BioLegend (11-26c.2a)	405750	1:1000
16	APCFire810-coupled anti-mouse B220	BioLegend (RA3-6B2)	103278	1:500
17	A700-coupled anti-mouse CD45.2	BioLegend (104)	109822	1:400
18	APC-e780-coupled Live/Dead	eBioscience	65-0865-14	1:10000

**Table 3:** List of antibodies used for fluorescence-activated cell sorting (FACS) of mouse cells

	Antibodies	Company and Clone	Identifier	Dilution
1	BUV661-coupled anti-mouse CD19	BD (1D3)	565076	1:500
2	BV510-coupled anti-mouse CD45.2	BioLegend (104)	109838	1:500
3	BV605-coupled anti-mouse IgG1	BD (A85-1)	563285	1:500
4	BV785-coupled anti-mouse B220	BioLegend (RA3-6B2)	103246	1:500
5	PerCPCy5.5-coupled anti-mouse CD38	BioLegend (90)	102722	1:500
6	PE-coupled NP	Biosearch Technologies	N-5070-1	1:100
7	PE-Cy7-coupled anti-mouse GL-7	BioLegend (GL-7)	144620	1:500
8	APC-coupled anti-mouse CD3	BioLegend (17A2)	100236	1:500
9	APC-coupled anti-mouse CD4	eBioscience (GK1.5)	17-0041-83	1:500
10	APC-coupled anti-mouse CD11c	BioLegend (N418)	117310	1:500
11	APC-coupled anti-mouse Ly6c	Invitrogen (HK1.4)	17-5932-80	1:500
12	A700-coupled anti-mouse CD45.1	BioLegend (A20)	110724	1:500
13	APC-e780-coupled Live/Dead	eBioscience	65-0865-14	1:10000

## Key Points

- Aged GC B cells can undergo positive selection in young mice upon immunisation.
- Aged GC B cells acquire high-affinity mutations post-immunisation in young mice.
- B cells from young mice have fewer high-affinity mutations in aged hosts.Introduction
