# Supplementary material for: B cells from aged mice do not have intrinsic defects in affinity maturation in response to immunisation
Source: J Immunol. Author manuscript; Available in PMC 2023 Nov 15. (PMC10627434; doi:10.4049/jimmunol.2300318)
Supplement: Supplementary figures [file EMS187608-supplement-Supplementary_figures.pdf]

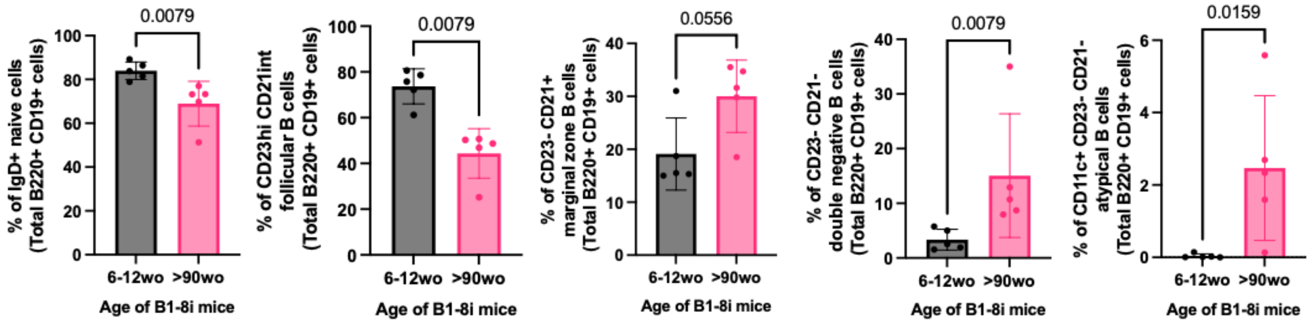


**Supplementary Figure 1. Lymph nodes of aged mice have reduced percentages of follicular B cells and increased proportions of atypical B cells.** Graphs depicting the percentage of IgD+ cells, follicular cells (CD23hi CD21int), marginal zone cells (CD23- CD21+), CD23- CD21- cells, and CD11c+ CD23- CD21- cells among B220+ CD19+ cells in young (6-12wo) and aged (90-100wo) B1-8i transgenic mice. Bar height corresponds to the mean, error bars indicate standard deviation, and each symbol represents values from individual mice. Statistics were calculated using unpaired Mann-Whitney U test. Data pooled from five independent repeat experiments.

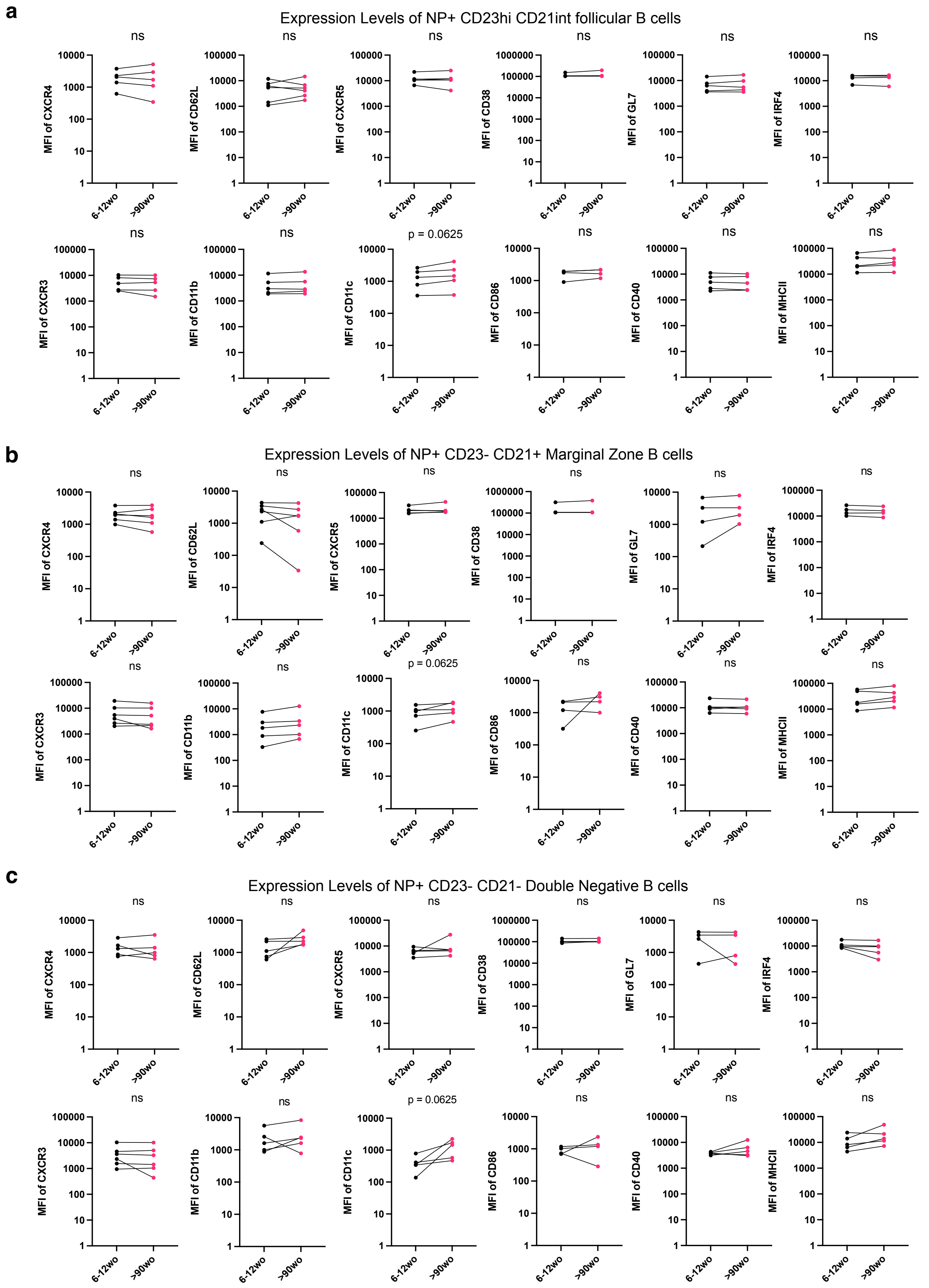


**Supplementary Figure 2. NP-specific B cell subsets from aged mice are phenotypically similar to those from young adult mice.** Graphs showing the mean fluorescence intensities (MFI) of CXCR4, CD62L, CXCR5, CD38, GL7, IRF4, CXCR3, CD11b, CD11c, CD86, CD40, and MHCII of NP+ follicular B cells **(a),** NP+ marginal zone B cells **(b)**, and NP+ CD21- CD23- B cells **(c)** from young (6wo) or aged (99wo) B1-8i transgenic mice. Each symbol represents values from individual mice, with young and aged donors from the same experiment shown as paired values. Statistics were calculated using Wilcoxon matched pairs signed rank test. Data pooled from at least four independent repeat experiments.

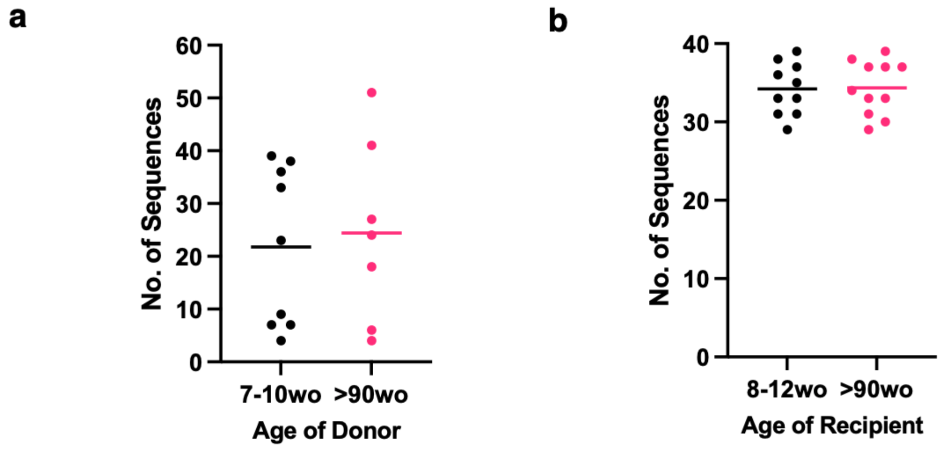


**Supplementary Figure 3.** Graphs showing the number of sequences analysed per recipient mice derived from sorted donor NP+ IgG1+ CD38- GL7+ B220+ CD19+ cells for the experiments in Figure 4 **(a)** and Figure 5 **(b)**.
